# On the effectiveness of random walks for modeling epidemics on networks

**DOI:** 10.1371/journal.pone.0280277

**Published:** 2023-01-10

**Authors:** Sooyeong Kim, Jane Breen, Ekaterina Dudkina, Federico Poloni, Emanuele Crisostomi

**Affiliations:** 1 Department of Energy, Systems, Territory and Constructions Engineering, University of Pisa, Pisa, Italy; 2 Faculty of Science, Ontario Tech University, Oshawa, Ontario, Canada; 3 Department of Computer Science, University of Pisa, Pisa, Italy; Federal University of Pernambuco: Universidade Federal de Pernambuco, BRAZIL

## Abstract

Random walks on graphs are often used to analyse and predict epidemic spreads and to investigate possible control actions to mitigate them. In this study, we first show that models based on random walks with a single stochastic agent (such as Google’s popular PageRank) may provide a poor description of certain features of epidemic spread: most notably, spreading times. Then, we discuss another Markov chain based method that does reflect the correct mean infection times for the disease to spread between individuals in a network, and we determine a procedure that allows one to compute them efficiently via a sampling strategy. Finally, we present a novel centrality measure based on infection times, and we compare its node ranking properties with other centrality measures based on random walks. Our results are provided for a simple SI model for epidemic spreading.

## 1 Introduction

### 1.1 Motivation

Markov chain models of dynamic processes on graphs have been exploited in a number of successful applications, of which the most notable one is perhaps Google’s PageRank algorithm [1]. Other successful applications include the modeling of power grids [2], connections of neurons in neuroscience [3], social networks [4], road networks [5], and epidemic networks [6].

These models rely upon the idea that a system may be modeled as a random walk, where the system transitions from state to state in discrete time-steps (possibly itself again) with a given probability that only depends on the current node of the system. In the aforementioned examples, this could be an Internet ‘surfer’ who chooses a link from one web-page to pass to the next web-page [7], or a car that at each intersection chooses the next road segment [5], or in electric circuits the incoming current at each junction is split in the out-going branches. In such examples, known parameters of graph theory and Markov chains may be used to infer interesting properties of the modeled case study, such as (for instance) mean first passage times, or the second-largest eigenvalue modulus, or Kemeny’s constant. For example, in [5], Kemeny’s constant is used as a measure of how well-connected the road network is, and the change in Kemeny’s constant upon removal of a road segment from the network is used as a measure of the importance of that road, thus using a well-understood Markov chain parameter associated with the network to make design and control decisions in the system.

Of particular interest is the consideration of spreading phenomena in a network: for example, the spread of disease in a contact network, the dissemination of rumours in an online social network, and so on. The starting point of this manuscript is the observation that a model based on a single random walker may not be strictly appropriate in such applications, and that one should interpret the properties of the random walk on a given graph carefully, rather than naively equating the behaviour of the random walk with the behaviour of the dynamical system it models.

In the literature, two main categories of random-walk models have been proposed to analyse epidemic networks. First, models based on a single random walker have been used, often derived from the expanding field of centrality measures [8]; they have been shown to provide interesting insight of the population networks; see, e.g., [6, 9, 10]. These models are particularly attractive because the relevant quantities are simple to compute also for relatively large networks. However, as we shall show in Section 3, these models fail to accurately predict the number of steps required to infect individuals in a network, as they do not consider the simultaneous presence of infected individuals in the network.

A second class of random-walk models includes models where several random walks occur simultaneously in the network, mimicking the actual dynamics of viruses and agents [11–13]. This approach fits into the general theory of agent-based methods: many variants have been proposed in the literature, sometimes with high degrees of sophistication; see, e.g., [14–18]. However, the math can be worked out to obtain closed formulas only in cases with particular topology or symmetries [19, 20]. In most cases, the relevant quantities can only be estimated through intensive Monte Carlo simulations, and can not be computed exactly using close formulas. Hence these models often suffer from scalability issues.

### 1.2 Contribution

In this manuscript we investigate random-walk based models for epidemic spreading and provide the following contributions to the state of the art:

1. First, we show through several examples why models based on a single random walker fail to accurately reproduce the dynamics of a virus in terms of infection times;
2. Second, we consider a classic 2^*n*^-state Markov chain SI model on a network, and establish closed-form expressions for computing relevant infection times. Such times are based on matrices that grow exponentially with the size of the population. Thus, we also provide an algorithm based on a sampling strategy that retains the actual mean infection times and is more computationally efficient than Monte Carlo simulations;
3. Finally, we propose a centrality measure called the *mean infection covering time* to rank nodes in a network by their influence in disease spread. Also, we compare it with other conventional indicators on several examples.

The paper is organized as follows: in Section 2, we give the mathematical formulation of Markov chains and random walks on graphs, along with the associated indicators that are used for quantitative and qualitative insights into systems modelled by these. In Section 3, we provide several motivating examples in which we compare naïve interpretations of random-walk parameters with simulated outcomes in the context of disease spread, in order to highlight some of the ways that this model does not capture the true dynamics. In Section 4, we describe a classic Markov chain model for disease spread which *does* effectively capture the disease dynamics. This model results in a Markov chain with 2^*n*^ states, as the state space describes the state (susceptible or infected) of every individual in the network with a binary representation. Such models do not scale efficiently with the size of the network, so we also provide an algorithm based on a sampling strategy to estimate mean infection times from one individual to another one, or from one group of individuals to another. In Section 5, we use the same concept to calculate the time for a single individual to infect the entire network, and propose these mean infection covering times (MICTs) as a centrality measure, and discuss how they compare with other random walk indicators that have been used in the literature for control measures such as vaccination or testing. Finally, in Section 6 we conclude our manuscript and outline interesting future lines of research.

## 2 Preliminaries

### 2.1 Graph theory

A graph *G* is a collection of vertices *V*, indexed *v*_1_, *v*_2_, …, *v*_*n*_, with a set of edges *E* consisting of pairs of vertices {*v*_*i*_, *v*_*j*_}. If {*u*, *v*} ∈ *E*, the vertices *u* and *v* are said to be *adjacent*; we also write *u* ∼ *v*, and say that *v* is a *neighbour* of *u*. The *degree* of a vertex *u*, denoted by deg(*u*), is the number of neighbours of *u*. The *adjacency matrix* of a graph is the matrix *A*(*G*) = [*a*_*i*,*j*_] such that


$$a_{i,j}=\{\begin{array}{cc} 1, & \;\text{if}\;v_{i}∼v_{j};\\ 0, & \;\text{otherwise.} \end{array}$$

Throughout this article, we consider only graphs which are simple (no loops or multiple edges between vertices), undirected (there is no orientation associated with an edge), and connected (for any pair of vertices *u* and *v*, it is possible to reach *u* from *v* via a sequence of adjacent vertices). We occasionally use ‘network’ and ‘graph’ interchangeably, and also substitute ‘vertex’ for ‘node’. The convention in the literature is that a *graph* refers to the abstract mathematical object, while a *network* is rooted in the real world in some way.

### 2.2 Markov chains

Suppose that we model a system as a stochastic process in which, at any given time, the system occupies one of a finite number of states *s*_1_, *s*_2_, …, *s*_*n*_, and transitions between these states in discrete time-steps with some fixed transition probabilities; that is, *t*_*i*,*j*_ denotes the probability of occupying *s*_*j*_ in the next time-step, given that the system is currently in *s*_*i*_. This may be represented as a sequence of random variables {*X*(*t*)∣*t* = 0, 1, 2, …}, indexed by time-step and taking values from the state space {*s*_1_, *s*_2_, …, *s*_*n*_}. Implicit in the description above is the so-called *Markov property*:

$$\begin{array}{cc} & P[X\left(k+1\right)=x_{k+1}|X\left(k\right)=x_{k},\ldots ,X\left(1\right)=x_{1},X\left(0\right)=x_{0}]\\ & \;=\;P[X\left(k+1\right)=x_{k+1}|X\left(k\right)=x_{k}]. \end{array}$$

Note that P[A∣B] denotes the conditional probability that event *A* occurs, given that event *B* occurs. The above re-states the assertion that the probability the system occupies a given state in the next time-step (at time *t* = *k* + 1) depends only on the current state of the system (at time *t* = *k*), and not on the state of the system in any previous step.

The probability transition matrix *T* = [*t*_*i*,*j*_] is central in the analysis of the behaviour of the Markov chain. The (*i*, *j*) entry of *T*^*k*^ gives the probability that the system occupies the state *s*_*j*_ of the chain after exactly *k* time-steps, given that the initial state was *s*_*i*_. Given an initial probability distribution vector **u** = [*u*_1_ *u*_2_ ⋯ *u_n_*]^⊤^ in which *u*_*i*_ is the probability of occupying *s*_*i*_ initially, the vector **u**^⊤^*T*^*k*^ gives the probability distribution across the state space at time *k*. Under certain conditions on the transition matrix *T* (primitivity), the Perron-Frobenius theorem indicates that as *k* → ∞, and independently of the initial distribution **u**, **u**^⊤^*T*^*k*^ converges to the unique stationary distribution ***π***^⊤^ of the Markov chain, which may be calculated as the unique left eigenvector of *T* corresponding to the eigenvalue 1, normalized so that the entries sum to 1. Since **u**^⊤^*T*^*k*^ represents the probability distribution at time *k*, this stationary distribution ***π*** represents the long-term probability distribution across the states; that is, *π*_*i*_ represents the probability of the system occupying the state *s*_*i*_ in the long run, or the proportion of time spent in *s*_*i*_.

To quantify the short-term behaviour of the system modelled by a Markov chain, we consider *mean first passage times*. For a Markov chain {*X*(*t*)∣*t* = 0, 1, 2, …}, the *first passage time* from *s*_*i*_ to *s*_*j*_ is the random variable *F*_*i*,*j*_ taking on the value of *t* for which *X*(*t*) = *s*_*j*_, given that *X*(0) = *s*_*i*_ and *X*(*k*) ≠ *s*_*j*_ for all *k* = 0, 1, …, *t* − 1. The mean first passage time, then, is the expected value of *F*_*i*,*j*_, denoted *m*_*i*,*j*_. While this definition is probabilistic in nature, it can be shown that the mean first passage times can be computed using the transition matrix *T*:

$$\begin{array}{c} m_{i,j}=\{\begin{array}{cc} e_{i}^{⊤}{\left(I-T_{\left(j\right)}\right)}^{-1}1, & i<j;\\ e_{i-1}^{⊤}{\left(I-T_{\left(j\right)}\right)}^{-1}1, & i>j. \end{array} \end{array}$$ (1)

Here **e**_*i*_ denotes the *i*^th^ standard basis vector, 1 the all-ones vector, and *T*_(*j*)_ denotes the principal submatrix of *T* obtained by deleting the *j*^th^ row and column. Here *m*_*i*,*j*_ is calculated as the row sum of (*I* − *T*_(*j*)_)^−1^ corresponding to *s*_*i*_. We note that in this article we take the convention that *m*_*i*,*i*_ = 0; alternatively we can define the *mean first return time* to *s*_*i*_, which can be shown to be equal to $\frac{1}{\pi_{i}}$. The *matrix of mean first passage times* is the matrix *M* = [*m*_*i*,*j*_], and while each entry can be computed as above, it is also well-known that

$$\begin{array}{c} M=\left(I-Z+JZ_{dg}\right)W^{-1}, \end{array}$$ (2)

where *Z* is the so-called *fundamental matrix* of the Markov chain (see [21]), *Z*_*dg*_ is the diagonal matrix whose entries consist of the diagonal entries of *Z*, and *W* is the diagonal matrix whose entries consist of the entries of ***π***.

Given an irreducible Markov chain with transition matrix *T*, stationary vector ***π***, and mean first passage matrix *M* = [*m*_*i*,*j*_], one can define, for a fixed index *i*, the quantity

$$\kappa_{i}=\sum_{j=1}^{n}\pi_{j}m_{i,j}.$$

This can be interpreted as the expected time to reach a randomly-chosen state *j*, starting from a fixed state *i*. Introduced in the 1960s in [21], this was remarkably shown to be independent of the choice of initial state *i*. As such, it is named Kemeny’s constant, and is denoted as K(T). Noting that $\pi^{⊤}1=1$, it can be shown that

$$\begin{array}{c} K\left(T\right)=\sum_{i=1}^{n}\sum_{j=1}^{n}\pi_{i}m_{i,j}\pi_{j}, \end{array}$$ (3)

admitting the interpretation of K(T) as the expected time of a random trip in the Markov chain, where the initial and terminal states of the trip are chosen at random, with respect to the stationary distribution.

A random walk on a given graph *G* = (*V*, *E*) is an example of a Markov chain. A random walker traverses the vertices of *G*, at each step choosing an adjacent vertex to move to uniformly at random. Thus the state space consists of the vertices *v*_1_, …, *v*_*n*_, and $t_{i,j}=\frac{1}{\text{deg}(v_{i})}$ whenever {*i*, *j*} ∈ *E*. Letting *D* be the diagonal matrix of vertex degrees, and *A*(*G*) the adjacency matrix of *G*, the probability transition matrix is given by *T* = *D*^−1^ *A*(*G*). Note that the stationary distribution vector is

$$\pi =\frac{1}{2|E|}{\left[\text{deg}(v_{1})\text{deg}(v_{2})\cdots \text{deg}(v_{n})\right]}^{⊤}.$$

Kemeny’s constant for the simple random walk on a graph *G* can be interpreted as a graph invariant indicating the ‘connectedness’ of the graph [5, 22], or how fast information ‘mixes’ in the graph [23, 24].

### 2.3 Absorbing Markov chains

A state *s*_*j*_ of a Markov chain is called *absorbing* if *t*_*jj*_ = 1; thus when the chain enters state *s*_*j*_, it remains there in every subsequent time-step. A Markov chain is called an *absorbing Markov chain* if its state space contains at least one absorbing state, and if for every *s*_*i*_ which is not absorbing, there exists some absorbing state *s*_*j*_ and some *k* > 0 such that $t_{i,j}^{\left(k\right)}>0$; that is, there is a nonzero probability that the Markov chain will reach one of the absorbing states in finite time, for any the initial state. While the analysis of irreducible Markov chains focuses on the limiting stationary distribution and mean first passage times, for absorbing Markov chains the limiting behaviour is always that the chain is eventually absorbed into one of the absorbing states, and one concerns oneself with the probabilities of absorption into the different absorbing states, and the expected time to absorption from some initial transient state.

Suppose the states are indexed so that the absorbing states are listed last in the ordering. This produces a block transition matrix as follows:

$$T=[\begin{array}{cc} Q & R\\ O & I \end{array}],$$

where *Q* is a square matrix representing transitions between transient states, *R* represents transitions from transient states to absorbing states, and *I* is the identity matrix, whose order is determined by the number of absorbing states. The analysis of the behaviour of the chain before absorption occurs centres around the computation of the matrix (*I* − *Q*)^−1^, the so-called *fundamental matrix for absorbing chains* (see [21]). Since

$${\left(I-Q\right)}^{-1}=I+Q+Q^{2}+\cdots ,$$

the (*i*, *j*) entry of this matrix captures the expected number of visits to the *j*^th^ transient state before absorption, given that the chain starts in the *i*^th^ transient state. Thus the *i*^th^ row of (*I* − *Q*)^−1^ *R* produces the probability distribution for the eventual absorbing state the chain ends up in, given that it starts in the *i*^th^ transient state, and the expected time to absorption given that the chain starts in the *i*^th^ transient state is computed as the *i*^th^ row sum of (*I* − *Q*)^−1^, or $e_{i}^{⊤}{\left(I-Q\right)}^{-1}1$.

**Remark 2.1.** Note that for an irreducible Markov chain, the expression for mean first passage times in (1) can be derived using these absorbing chain techniques described above, by designating the state *s*_*j*_ as an absorbing state and replacing the *j*^th^ row of *T* by zeros with a 1 in the *j*^th^ position. Then the mean first passage times can be computed as the expected time to absorption, where the matrix *Q* is the principal submatrix *T*_(*j*)_. Furthermore, if one requires the expected time to reach a collection J of states, this can be determined by the appropriate row sum of ${\left(I-T_{\left(J\right)}\right)}^{-1}$, where by $T_{\left(J\right)}$ we denote the principal submatrix of *T* with the rows and columns indexed by J removed. This is a key observation which we will use to calculate mean infection times in the 2^n^-state model in Section 4. This result is well-known in the literature; see for example the discussion found in [25].

### 2.4 Epidemic modelling

In this article our focus is on exploring the ability of Markov chain models to reflect the dynamics of virus spreading in populations. For this purpose we shall only consider elementary SI epidemic models for the disease, where individuals may either be susceptible (S) or infected (I); and once an individual is infected, it remains infected indefinitely [26]. If two individuals are in contact and one is susceptible and the other infected, there is a fixed infection probability *β* that the susceptible individual also becomes infected. Throughout this work, when simulations are done we assume the infection probability is *β* = 0.1.

Compartmental models of disease spread such as SI models traditionally make the assumption that the population is *well-mixed*, which means that every individual comes into contact with every other individual (making *β* the rate of transmission). In a network representation of the population, each individual is represented by a node, and an infected individual may infect another susceptible individual in the next time-step if the two of them are adjacent. This underlying graph is a contact network determining which individuals in the population have been in close contact that is sufficient to facilitate the spread of disease, and this network can be inferred from data in some way (see for example [27]).

Given a connected graph and at least one initially-infected individual, at some point all of the individuals in the network will be infected as well. That is, the equilibrium of the model is the state in which every individual is infected. The focus of our analysis of the above model of epidemics is on quantities dictating how fast the disease will spread. By considering quantities such as the time until the entire network is infected (i.e. until equilibrium is reached), or the time until a particular individual becomes infected, we can better measure and understand the role of an individual node in the dynamical process. We note that these concepts may apply naturally to other domains such as fake news spreading in online social networks, as similar epidemic models have been used in [28, 29] regarding such applications.

## 3 Infection time predictions for single random walk models

**Fig 1:**
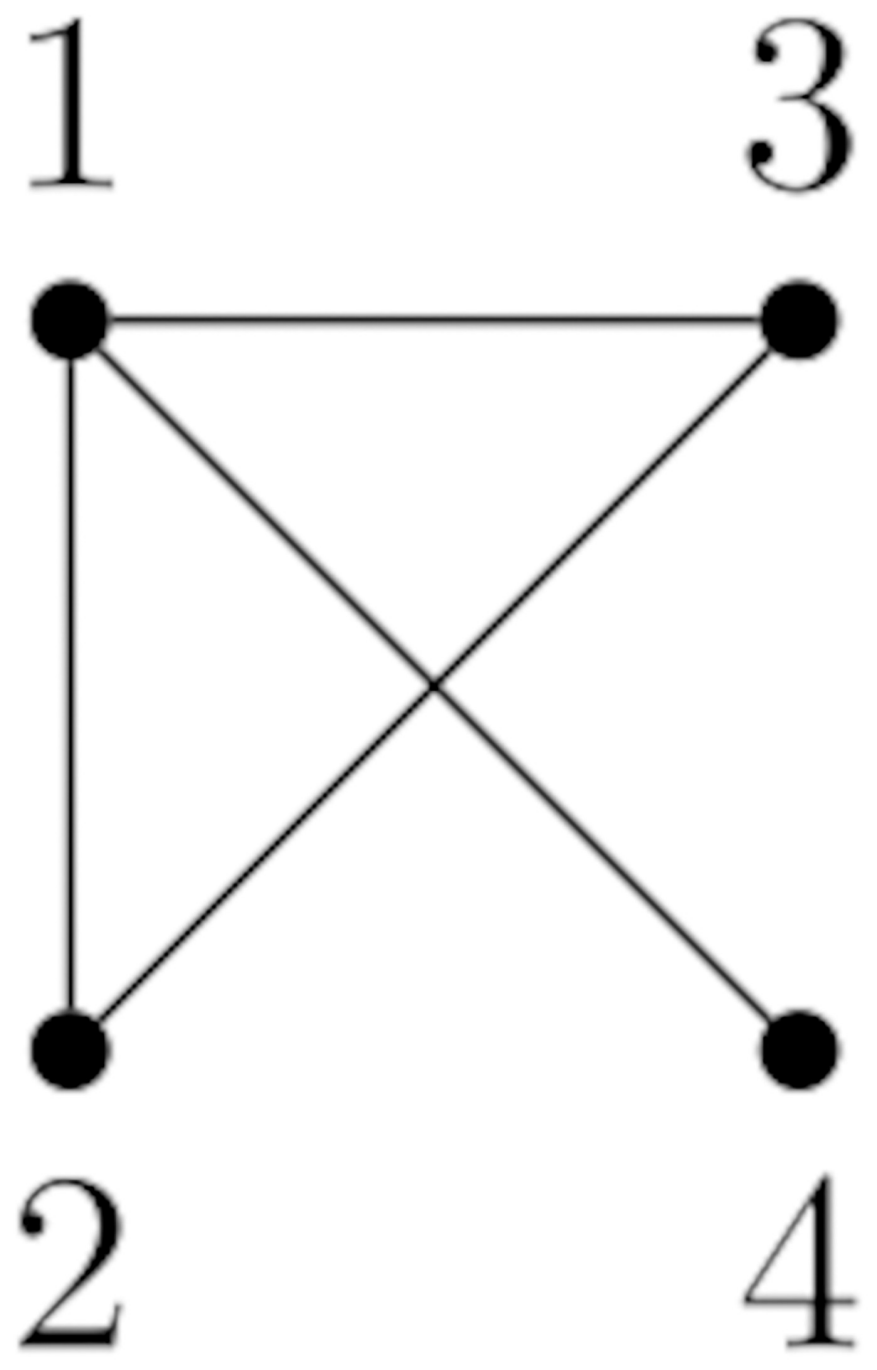
A graph with 4 nodes used in Section 3 as a simply clarifying example.

In this section, we show that there is a discrepancy between the infection times predicted by random walk models with single walkers and the actual times observed in SI models. We first consider the graph in Fig 1 as a supporting example to clarify our discussion.

The probability transition matrix for the simple random walk on this graph is

$$\begin{array}{c} T_{RW}=[\begin{array}{cccc} 0 & 1/3 & 1/3 & 1/3\\ 1/2 & 0 & 1/2 & 0\\ 1/2 & 1/2 & 0 & 0\\ 1 & 0 & 0 & 0 \end{array}]. \end{array}$$

In comparing this random walk with the movement of disease, there are some issues to note. If the (*i*, *j*) entry of *T*_*RW*_ is naïvely interpreted as the probability of the disease spreading from individual *i* to individual *j*, then immediately we see that a contact of the same duration between individuals 1 and 4 produces two different probabilities *t*_14_ and *t*_41_, due to the different degrees of the two nodes.

To overcome the problems of having contact of the same durations associated with different probabilities of spreading the virus, we can consider modifying the transition matrix to create a symmetric one, which we denote *T*_*sym*_, adapting the example of [30]. The following definition can be used to account for the infection probability and constructs a symmetric probability transition matrix, so that the probability of *i* to infect *j* is the same as that of *j* to infect *i*. In fact, the matrix is defined so that every off-diagonal entry is equal. This matrix is necessarily doubly-stochastic (both the rows and columns sum to 1). We define

$$\begin{array}{c} T_{sym}=\alpha A\left(G\right)+(I-\alpha D), \end{array}$$

for some $0<\alpha \leq \frac{1}{d_{\text{max}}}$ where *d*_max_ is the maximum node degree, and *D* is the diagonal matrix of vertex degrees. Note that *αA*(*G*) is a substochastic matrix with zero diagonal, while *I* − *αD* is a diagonal matrix. One can interpret this definition as a transition matrix for a random walk on a graph where, at each vertex, the random walker has some fixed constant probability *α* of choosing any of the neighbours of the current vertex, but with the residual probability can choose instead to remain in place. For the graph in Fig 1, choosing *α* = 0.1, we have

$$\begin{array}{c} T_{sym}=[\begin{array}{cccc} 0.7 & 0.1 & 0.1 & 0.1\\ 0.1 & 0.8 & 0.1 & 0\\ 0.1 & 0.1 & 0.8 & 0\\ 0.1 & 0 & 0 & 0.9 \end{array}]. \end{array}$$

**Remark 3.1.** We note that in this example, every off-diagonal entry is equal to the infection probability *β* = 0.1 that we wish to use in the disease model. In the case that the desired infection probability is larger than $\frac{1}{d_{\text{max}}}$, it is not possible to choose *t*_*ij*_ = *β* for transition probabilities between individuals joined by an edge. However, we remark here that mean first passage times between distinct states for transition matrices of this form scale in a natural way with varying choices of *α*. That is, if *T*_1_ = *α*_1_ *A*(*G*) + (*I* − *α*_1_ *D*) and *T*_2_ = *α*_2_ *A*(*G*) + (*I* − *α*_2_ *D*), then for *i* ≠ *j*, $m_{i,j}^{\left(T_{1}\right)}=\frac{\alpha_{2}}{\alpha_{1}}m_{i,j}^{\left(T_{2}\right)}$. Thus for the sake of *proportional* comparison of mean first passage times with the infection times obtained from simulations, the value of *α* is irrelevant.

We now intend to uncover an inaccuracy of the models based on random walks. For this purpose, we compute the mean first passage times *m*_*i*,*j*_ for a random walk on the graph. To compare, we also extensively simulate the spreading of the virus, and compute from simulations the average time *M*_*i*,*j*_ for individual *j* to be infected, given that we start with only individual *i* infected. These are the values which are compared in order to examine the efficacy of random-walk-based methods.

Let *M*_*RW*_ and *M*_*sym*_ be the matrices of MFPTs for the random walk and the symmetric random walk on the graph in Fig 1, respectively. Then, *M*_*RW*_ and *M*_*sym*_ can be computed using (1) or (2) to produce:

$$\begin{array}{c} M_{RW}=[\begin{array}{cccc} 0 & 3.3333 & 3.3333 & 7\\ 2 & 0 & 2.6667 & 9\\ 2 & 2.6667 & 0 & 9\\ 1 & 4.3333 & 4.3333 & 0 \end{array}],\;\;M_{sym}=[\begin{array}{cccc} 0 & 16.6667 & 16.6667 & 30\\ 10 & 0 & 13.3333 & 40\\ 10 & 13.3333 & 0 & 40\\ 10 & 26.6667 & 26.6667 & 0 \end{array}]. \end{array}$$

On the other hand, we can estimate the time for an initially-infected individual *i* to cause individual *j* to become infected using simulations. Assuming that the probability of spreading the virus is equal to 0.1, we ran 100000 Monte Carlo simulations with Algorithm 1. We estimated a matrix *M_inf_* of these expected times regarding infection as follows:

$$\begin{array}{c} M_{inf}\approx [\begin{array}{cccc} 0 & 7.7922 & 7.7766 & 10.0051\\ 7.7503 & 0 & 7.7358 & 17.7442\\ 7.7383 & 7.7284 & 0 & 17.7707\\ 10.0059 & 17.7454 & 17.7628 & 0 \end{array}]. \end{array}$$ (4)

**Algorithm 1:** Monte Carlo estimation of *M_inf_* = [*M_i,j_*]

**Data**: A graph *G* = (*V*, *E*); infection probability *β*; a number of iterations *N*.

*M*_*inf*_ ← 0;

**for** *k* = 1, 2, …, *N* **do**

 **for** *i* ∈ *V* **do**

  **x** ← *e_i_*; *t* ← 0;

  **while** x≠1 **do**

   *t* ← *t* + 1;

   **for** {*v*_*l*_, *v*_*j*_} ∈ *E* *with* *x*_*l*_ = 1 *and* *x*_*j*_ = 0 **do**

    Assign 1 to *x*_*j*_ with probability *β*;

    **if** *x*_*j*_ = 1 **then**

$M_{i,j}^{\left(k\right)}\leftarrow t$ ;

    **end**

   **end**

  **end**

 **end**

 *M*_*inf*_ ← *M*_*inf*_ + *M*^(*k*)^;

**end**

*M*_*inf*_ ← *M*_*inf*_/*N*;

Apart from scaling factors (one could normalize *M*_*RW*_ or *M*_*sym*_ for comparison purposes), it is still obvious that there is a disparity between the estimated values and the theoretical mean first passage times associated with the simple or symmetric random walks. Different predictions are obtained in general; as a single example, the ratio of *m*_1,3_ to *m*_1,4_ in *M*_*RW*_ and the ratio of the corresponding entries in *M*_*sym*_ are both close to 2, while the ratio of the corresponding entries in *M*_*inf*_ is close to 1.

**Fig 2:**
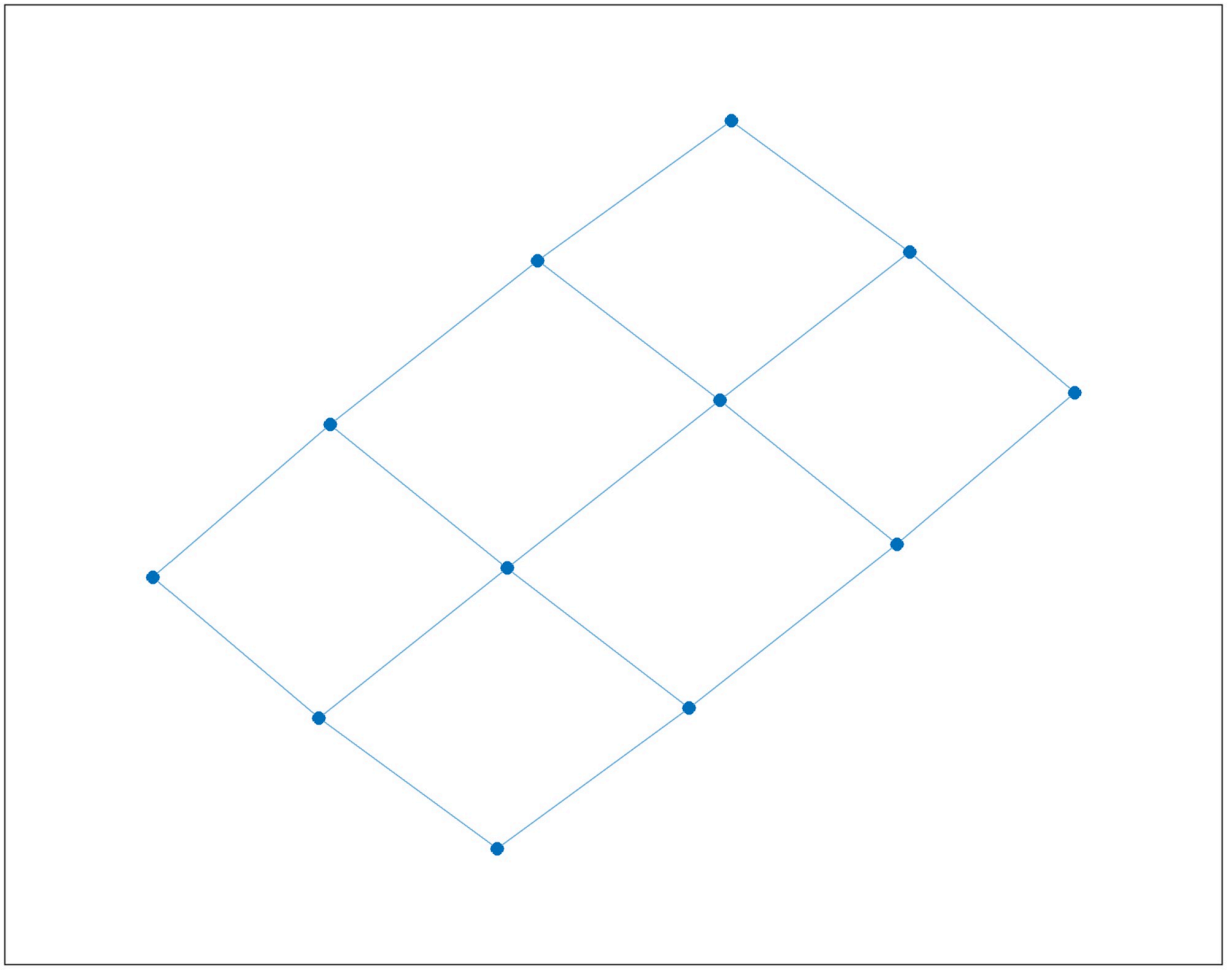
The graph *L*(3, 4).

**Fig 3:**
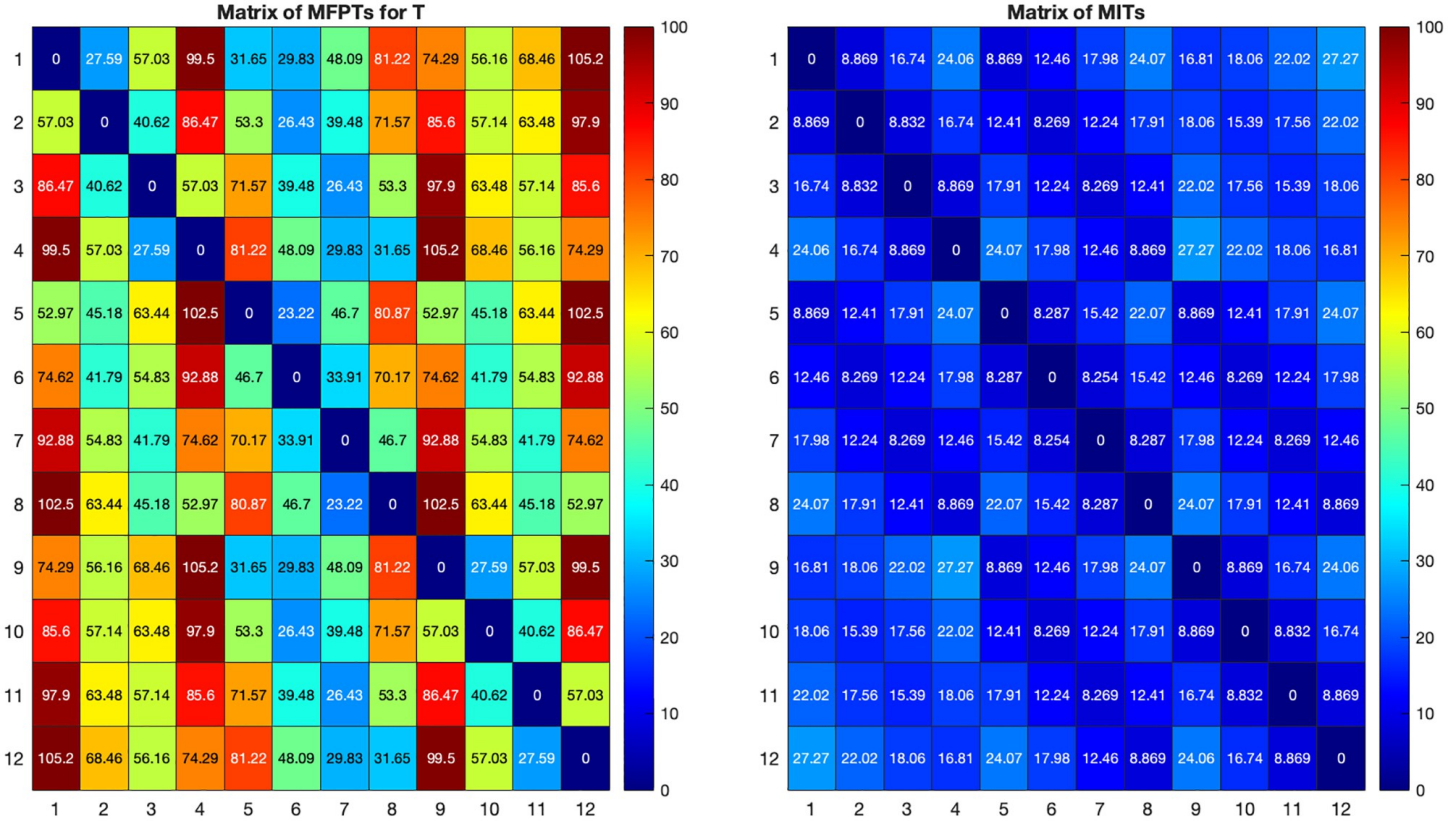
Comparison of mean first passage times for *T* and mean infection times in *L*(3, 4).

We consider another example which arises in the context of traffic flow in urban road networks, another domain of application in which random walks have been shown to be an effective model. Let *L*(*m*, *n*) denote an *m* × *n* lattice graph or grid graph; see Fig 2. We now consider a random walk on this lattice, under the assumption that the next state is chosen among the available neighbours with the same probability *p* = 0.1, while with the residual probability the state will not change (i.e., the random walker stays in place). For instance, this may correspond to a vehicle travelling in a lattice-like road network, where edges correspond to roads and nodes to intersections. At each intersection, the car will choose the next road with the same probability 0.1, and with the residual probability will remain in the same place. For example, for nodes in the middle of the lattice *L*(3, 4) in Fig 2 which have 4 neighbours, this corresponds to saying that with probability 0.6 the state will not change in one step, and with probability 0.4 it will change state, with the same probability 0.1 of choosing any one of the four neighbours. In this case, it was shown in [5] that the mean first passage times (MFPTs) to travel from any state to any other state according to such a random walk, can be computed by using the transition matrix *T* given by *T* = 0.1*A*(*G*) + (*I* − 0.1*D*) where *G* is *L*(3, 4). The values of such MFPTs are depicted on the left of Fig 3.

We now consider the same graph, but we assume now that the graph depicts the social interactions between individuals, and we compute how long it would take for a virus to spread from individual *i* to individual *j* in reality. We assume again that the probability of spreading the virus in one step is *β* = 0.1, but in this context, one individual can infect more than one neighbour in a single step. Accordingly, the mean time to infection from one individual to any other individual are shown on the right of Fig 3. Mean infection times (MITs) from *i* to *j* are computed using Algorithm 1 under the assumption the *i* is the only infected individual at the first time-step, and it is straightforward to see that such times are much smaller than those that would have been estimated through a conventional random walk model.

Another example that displays clearly how MFPTs and MITs are qualitatively different is the following.

**Fig 4:**
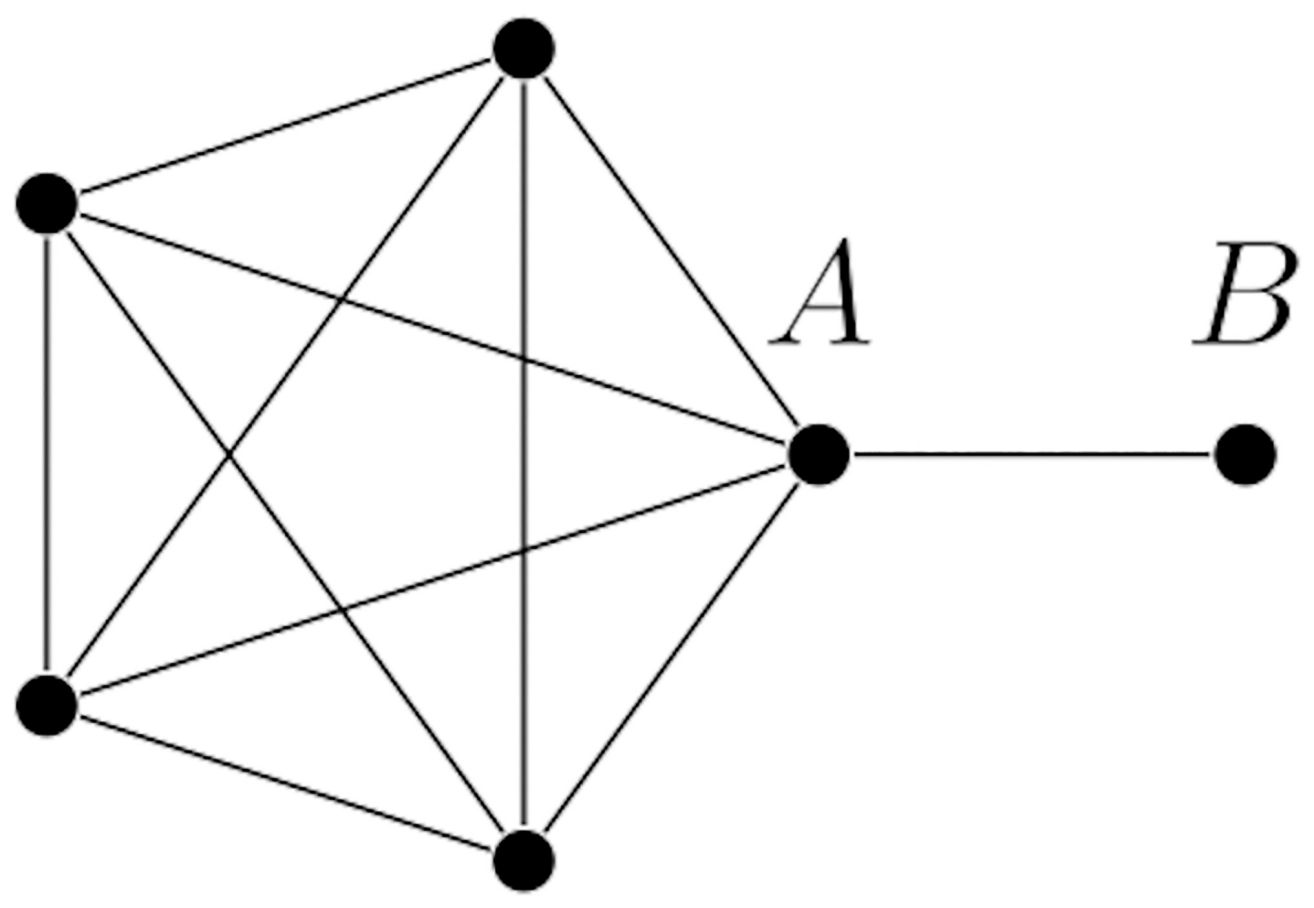
A graph constructed from a complete graph on *k* = 5 nodes by adding a new node.

**Example 3.2.** Consider a complete graph on *k* nodes, and to one of these nodes (which we shall call *A*) attach a new node (which we shall call *B*). An example with *k* = 5 is depicted in Fig 4.

We shall show that the MIT from node *A* to *B* is constant, while the MFPT between the same two nodes grow quadratically with *k*.

Suppose that *A* is the only initially infected individual. Then, at each time-step *B* has a probability *β* of becoming infected, hence the MIT from *A* to *B* is the expected value of a geometric distribution with parameter *β*, that is, $E\left[\text{Geo}\left(\beta \right)\right]=\frac{1}{\beta }$. This probability is independent of *k*. On the other hand, the mean time needed for a random walk starting from *A* to reach *B* increases as *k* grows: indeed, for large *k*, the random walk on the clique takes more time to return to *A*, since there are more nodes available to visit. More precisely, let *M* be the mean first passage time from *A* to *B*. Starting from *A*, the random walk either reaches *B* immediately in time 1, with probability $\frac{1}{k}$, or moves to another node of the clique with probability $\frac{k-1}{k}$. Then, at each subsequent time-step, it has probability $\frac{1}{k-1}$ to return to *A*, hence the time of first return to *A* from another node of the clique is $E[\text{Geo}\left(\frac{1}{k-1}\right)]=k-1$. After the process has returned to *A*, by the Markov property we need again an average time *M* to reach *B*. This argument produces the equation $M=\frac{1}{k}1+\frac{k-1}{k}\left(1+\left(k-1\right)+M\right)$, which can be solved for *M*. Hence, *M* = *k*^2^ − *k*+ 1.

### 3.1 Discussion

Even in very simple examples, it is obvious to see that modelling the spreading of the virus in a population as a random walk with a single agent leads to some coarse approximations in terms of the expected mean first passage times. This is rather important to note, as many researchers have used indicators based on random walks, and MFPTs explicitly, in a number of epidemic applications (e.g., vaccination or testing strategies). For instance, this comment holds for indicators such as Kemeny’s constant [6], or the random walk centrality (RWC) [31]. Also, random walk betweenness (RWB), originally introduced by Newman [32] is a classic indicator based on random walks that is known to be one of the best indicators for which individuals should be vaccinated in a population [33].

All such indicators may still provide interesting insights in the dynamics of a virus based on the underlying structure of the contact network—for example, in [6] it is shown that Kemeny’s constant can be used to detect bridges between disparate communities in contact networks. The effectiveness of these indicators lies in the fact that they are obviously based on the topological structure of a population and of its average contacts; still, one may wonder about the impact of decisions that could be made on more precise models of the disease dynamics, rather than simply the network structure which is revealed through random walk methods. While we do not come to a conclusive answer to that question in this article, we provide the tools by which one can appropriately model and measure the disease spread using a different Markov chain model, and we explore some examples for which random walk indicators disagree with those determined from the more intuitive model.

## 4 Exact Markov chain models and efficient algorithms to compute mean infection times

We suggest that the primary reason that a random walk seems to be ineffective as a model for disease spread in a network is that previously-infected individuals remain infectious throughout the process, continuing to infect their contacts and affecting the overall dynamics of the disease in future time-steps. Even multi-agent systems modelled with multiple random walkers will not accurately capture this aspect of the disease spread context. As such, we require a model which retains information about every individual’s status at once. In this section, we describe such a model which is known in the literature, and which has been used in a variety of settings and in many forms. We will review some of these results, and our contribution to the literature is to then define and investigate an analogue of mean first passage times in this setting. We determine how to compute these exactly (the values which were simulated in Section 3), and discuss some computational issues, as well as determine an algorithm in Sections 4.2 and 4.3 to effectively approximate these in the case that it is computationally expensive to compute exactly. We then proceed in Section 5 to establish how these quantities can be developed to determine a centrality indicator for use in control measures in disease spread settings.

### 4.1 2^*n*^-state model of epidemic dynamics

Let *G* = (*V*, *E*) be a graph, and suppose its vertices are labelled *v*_1_, *v*_2_, …, *v*_*n*_. Let **X**(*t*) = (*X*_1_(*t*), …, *X*_*n*_(*t*)) ∈ {0, 1}^*n*^ where

$$X_{i}\left(t\right)=\{\begin{array}{cc} 1, & \text{if}\;\text{node}\;v_{i}\;\text{is}\;\text{infected}\;\text{at}\;\text{time}\;t;\\ 0, & \text{otherwise.} \end{array}$$

Since we consider the SI model, we assume that the probability of recovery is 0; hence if *X*_*i*_(*t*_0_) = 1, then *X*_*i*_(*t*) = 1 for all *t* > *t*_0_ (models with nonzero recovery probabilities are used in [34–36]). Assume that in any contact between a susceptible individual and an infected individual, the probability of infection is some constant *β*. Then, {**X**(*t*)∣*t* = 0, 1, …} is a discrete-time, time-homogeneous Markov chain with a finite state space *S* = {0, 1}^*n*^; that is, each state is represented by a binary vector or *n*-tuple in which the *k*th entry is 1 if *v*_*k*_ is infected, and 0 if *v*_*k*_ is suspectible.

For a subset *A* ⊂ *V*, we denote by *s*_*A*_ the *n*-tuple in *S* whose *k*^th^ component is 1 if *v*_*k*_ ∈ *A*, and zero if *v*_*k*_ ∉ *A*. For simplicity, we write *s*_{*v*_*i*_}_ as *s*_*i*_. In what follows, we assume that $P\left[X\left(t+1\right)=s_{i}|X\left(t\right)=\left(0,\ldots ,0\right)\right]=\frac{1}{n}$ for 1 ≤ *i* ≤ *n*, so that if initially no one is infected, an individual is chosen uniformly at random to be infected in the next step. Then, {**X**(*t*)} is an absorbing Markov chain with exactly one absorbing state *s*_*V*_ = (1, …, 1).

Suppose that node *v*_*i*_ is susceptible, and has *n*_*i*_ infected neighbours at time *t*. Since the infections are independent, the probability that *v*_*i*_ is not infected at time *t* + 1 is ${\left(1-\beta \right)}^{n_{i}}$. Hence

$$\begin{array}{cc} \mathbb{P}\left[X_{i}\left(t+1\right)=y_{i}|X\left(t\right)=x\right]= & \left\{\begin{array}{ll} 1, & \text{if}\;yi=xi=1;\\ 1-{\left(1-\beta \right)}^{n_{i}}, & \text{if}\;yi=1,\;xi=0;\\ {\left(1-\beta \right)}^{n_{i}}, & \text{if}\;yi=0,\;xi=0;\\ 0, & \text{if}\;yi=0,\;xi=1. \end{array}\right. \end{array}$$ (5)

Then, again by the independence of infections, for each **x**, **y** ∈ *S*, the transition probability from **x** to **y** is given by

$$\begin{array}{c} P\left[X\left(t+1\right)=y|X\left(t\right)=x\right]=\prod_{i=1}^{n}P\left[X_{i}\left(t+1\right)=y_{i}|X\left(t\right)=x\right] \end{array}$$ (6)

We note that if **y** does not have at least the same infected population as **x**, then P[X(t+1)=y∣X(t)=x]=0. This means that the states can be ordered in such a way that the probability transition matrix *T* is an upper triangular matrix.

We now provide a method to obtain the expected time for a group of some infected individuals to infect—directly or indirectly—another group of susceptible individuals. That is, given a subset of individuals *A* who are initially infected, we compute the expected time until a subset *B* of individuals are infected, where *B* ⊃ *A*. If we choose *A* = {*v*_*i*_} and *B* = {*v*_*i*_, *v*_*j*_}, this enables us to exactly compute the matrix *M*_*inf*_ in (4). We define terminology for such expected times in order to distinguish from the mean first passage times used in the previous sections.

**Definition 4.1.** Let *G* = (*V*, *E*) be a connected graph, and let *A*, *B* ⊆ *V* with *A* ⊂ *B*. The *mean infection time* (MIT) from *A* to *B*, denoted as *μ*_*A*, *B*_, is the expected time for all nodes in *B*\*A* to get infected, given that all nodes in *A* are initially infected. As an analogous concept to the mean first passage matrix, we define the *MIT matrix* to be the matrix *M*_*inf*_ = [*M*_*i*,*j*_] where $M_{i,j}=\mu_{\left\{v_{i}\},\{v_{i},v_{j}\right\}}$.

**Definition 4.2.** Let *G* = (*V*, *E*) be a connected graph, and let *u* ∈ *V*. The *mean infection covering time* (MICT) of *u*, denoted *m*_*u*_, is the mean infection time from *A* = {*u*} to *B* = *V*; that is, *m*_*u*_ = *μ*_{*u*}, *V*_, and represents the expected time until the entire network is infected, given that *u* is the only initially-infected node.

We now give a closed form expression for *μ*_*A*, *B*_, for *A*, *B* ⊆ *V* with *A* ⊂ *B*. Let us consider the set *S*_*B*_ ⊆ *S* that consists of binary vectors with ones in the positions corresponding to *B*; that is, states in which at least the vertices corresponding to *B* are infected, and possibly others infected too. Then, the mean infection time from *A* to *B* is the expected value of the first time (hitting time) at which the system arrives at one of states in *S*_*B*_, starting from the state *s*_*A*_. This is the mean first passage time in the Markov chain with 2^n^ states, from the state *s*_*A*_ to the *collection* of states *S*_*B*_. As discussed in Remark 2.1, we have

$$\begin{array}{c} \mu_{A,B}=e_{s_{A}}^{⊤}{\left(I-T_{\left(S_{B}\right)}\right)}^{-1}1 \end{array}$$ (7)

where $e_{s_{A}}$ is the column vector whose entry corresponding to the state *s*_*A*_ is 1 and zeros elsewhere; *T*_(*S*_*B*_)_ is the principal submatrix of *T* obtained by removing rows and columns corresponding to states in *S*_*B*_; and 1 is the all-ones vector.

This general expression can be used to calculate the entries of the MIT matrix for a given network, and can be used to calculate the mean infection covering times with the set *A* representing a single vertex, and *B* is the entire vertex set. The remainder of this article focuses on these two cases, but there are many uses for (7) in the context of disease spread and other spreading processes. For example, by setting *S*_*B*_ to be the set of all binary vectors with more than *p*% ones (i.e. states in which more than *p*% of individuals are infected), and *A* to be some initially-infected set of interest, we can determine the time until some threshold of infections is reached (for some diseases, this is of interest for herd immunity).

Due to the increased size of the state space (from *n* states to 2^n^ states, where *n* is the size of the population), the calculation of these mean infection times becomes computationally expensive. In the remainder of this section, we discuss some computing strategies for the exact computation, then introduce and discuss approximation strategies in Sections 4.2 and 4.3, respectively.

We first note that we need not consider the entire matrix $T_{\left(S_{B}\right)}$ for finding *μ*_*A*, *B*_. In an SI epidemic model, for any state **x** such that *x*_*k*_ = 0 for some *k* ∈ *A*, the transition probability from *s*_*A*_ to **x** is 0. That is, if *S*_*A*_ is the set of states consisting of binary vectors with ones in the positions corresponding to *A*, and ${\bar{S}}_{A}=S\backslash S_{A}$ is its set complement, then if the initial state is *s*_*A*_ we may remove from consideration any states of the Markov chain from ${\bar{S}}_{A}$ without affecting the outcome of the computation. These correspond to states in which some individual who was initially infected is now not infected, which is impossible in the SI model. Hence, instead of $T_{\left(S_{B}\right)}$ in (7), we may use the matrix $T_{\left({\bar{S}}_{A}\cup S_{B}\right)}$ obtained from *T* by deleting rows and columns corresponding to the states in ${\bar{S}}_{A}\cup S_{B}$. This substochastic matrix has rows and columns indexed by the states in *S*_*A*_. Therefore,

$$\begin{array}{c} \mu_{A,B}=e_{s_{A}}^{T}{\left(I-T_{\left({\bar{S}}_{A}\cup S_{B}\right)}\right)}^{-1}1. \end{array}$$ (8)

Note that $e_{s_{A}}$, *I* and 1 are re-sized appropriately (their size determined in context), and that $e_{s_{A}}$ is the standard basis vector with a 1 in the position corresponding to the state *s*_*A*_, according to where it appears in the reduced state list.

Now let us consider a mean infection covering time *μ*_{*v*_*i*_}, *V*_. The size of the matrix *T* is exponential, so it is impractical to store data for *T* when the number of nodes in the corresponding graph is sufficiently large. Note that *T* is an upper triangular matrix, and computational experiments show that the sparsity of *T* is between 1% and 5% for Erdős–Rényi random graphs on 10 nodes with probability 0.5. Thus one could consider using a Sparse Triangular Matrix Solver (SpTrSV) (see [37] for a brief introduction and algorithms). In order to implement SpTrSV, it requires particular data formats [38] such as the compressed sparse row (CSR) or the compressed sparse column (CSC), which are two formats for storing nonzero entries of a sparse matrix into three row vectors. However, as the size of the matrix increases exponentially, the number of nonzero entries would increase exponentially since the graphs we consider are connected. Hence, SpTrSV would also eventually become infeasible for the computation of mean infection covering times as in (10).

In order to see how using the transition matrix *T* might not be practical, we consider a particular example of a tree, which is a minimally-connected graph (that is, it has the least number of edges necessary to be a connected graph). Let *G* be a star with vertex set {1, …, *n*}—that is, one vertex is of degree *n* − 1 and the others have degree 1. Suppose that vertex 1 is of degree 1; and vertex 2 is of degree *n* − 1. Assume that *X*(0) = (1, 0, …, 0) and consider the 2^*n*−1^ × 2^*n*−1^ submatrix of the transition matrix. It can be seen that there are 2^*n*−2^ + 1 nonzero entries on the off-diagonal. In empirical settings, the storage of data like this becomes an issue. Even though *G* is one of the sparsest graphs, we may not be able to calculate explicitly our desired mean infection times for sufficiently large orders of *G* by using (10).

**Example 4.3.** Consider the graph *G* in Fig 1. Let *V* = {*v*_1_, *v*_2_, *v*_3_, *v*_4_}, *A* = {*v*_1_}, and *B* = {*v*_1_, *v*_2_}. For simplicity, we shall remove parentheses and commas of all states in *S*. Then, *s*_*A*_ = 1000, *S*_*A*_ = {1000, 1100, 1010, 1001, 1110, 1101, 1011, 1111}, and *S*_*B*_ = {1100, 1110, 1101, 1111}. One can verify that we obtain the matrix

$$\begin{array}{c} T_{\left({\bar{S}}_{A}\cup S_{B}\right)}=[\begin{array}{cccc} 0.729 & 0.081 & 0.081 & 0.009\\ 0 & 0.729 & 0 & 0.081\\ 0 & 0 & 0.81 & 0.09\\ 0 & 0 & 0 & 0.81 \end{array}]. \end{array}$$

This substochastic matrix represents transitions between all possible states in which *v*_1_ is infected, but *v*_2_ is not yet infected. Rows and columns correspond to the states 1000, 1010, 1001, 1011, in that order. Note that these states correspond to the set *S*_*A*_\*S*_*B*_. From (8), we have *μ*_*A*, *B*_ = 7.7562 (rounded to four decimal places), which is the expected time for susceptible individual 2 to get infected, provided the only infected node is 1. So, *μ*_*A*, *B*_ corresponds to the (1, 2) entry of *M*_*inf*_ in (4). In this manner, one can calculate the matrix *M*_*inf*_ (rounded up to 4 decimal places) as follows:

$$\begin{array}{c} M_{inf}=[\begin{array}{cccc} 0 & 7.7562 & 7.7562 & 10\\ 7.7562 & 0 & 7.7562 & 17.7562\\ 7.7562 & 7.7562 & 0 & 17.7562\\ 10 & 17.7562 & 17.7562 & 0 \end{array}]. \end{array}$$ (9)

Moreover, one can also find the MICTs: $m_{v_{1}}=14.3534$, $m_{v_{2}}=m_{v_{3}}=18.5014$, and $m_{v_{4}}=20.2493$. This suggests that *v*_1_ is the most critical node in the sense that a single individual quickly spreads the virus to the whole network.

### 4.2 A sampling strategy for the 2^n^-state model

In this subsection, we give an alternative construction of the 2^n^-state model, which allows us to use a sampling method for more efficient approximation of the mean infection times, and can be used to prove a nontrivial symmetry property. This construction is a discrete-time analogue of the continuous version appearing in [39, Theorem II.2], which the authors describe as ‘folklore in some circles’.

Let us consider a single edge {*v*_*i*_, *v*_*j*_} ∈ *E* in the network. Assuming that one of its two endpoints is infected and the other is not, the time *τ*_*i*,*j*_ taken for the infection to spread across the edge follows a geometric distribution, i.e., $P\left[\tau_{i,j}=k\right]={\left(1-\beta \right)}^{k-1}\beta$ for each *k* = 1, 2, …. We call *τ*_*i*,*j*_ the *potential infection time* over the edge *i*, *j*, because we are working under the assumption that the infection spreads over this edge of the network from one of its endpoints to the other.

In this new alternative construction, we first assign independently a value *τ*_*i*,*j*_ to each edge {*v*_*i*_, *v*_*j*_} ∈ *E*, sampling according to the geometric distribution described above. Once these values *τ*_*i*,*j*_ have been chosen, we can reconstruct the dynamics of the infection starting from an initial infected set $\hat{X}\left(0\right)=s_{A}$ for some *A* ⊆ *V*: an infection happens across edge {*v*_*i*_, *v*_*j*_} after time *τ*_*i*,*j*_ if one of the two endpoints is infected and the other is not. Hence the infection spreads from a vertex *v*_*k*_ to another vertex *v*_ℓ_ in time *t* equal to the graph distance dist(*v*_*k*_, *v*_ℓ_) on the graph *G*, with weights (edge lengths) given by *τ*_*i*,*j*_.

**Fig 5:**
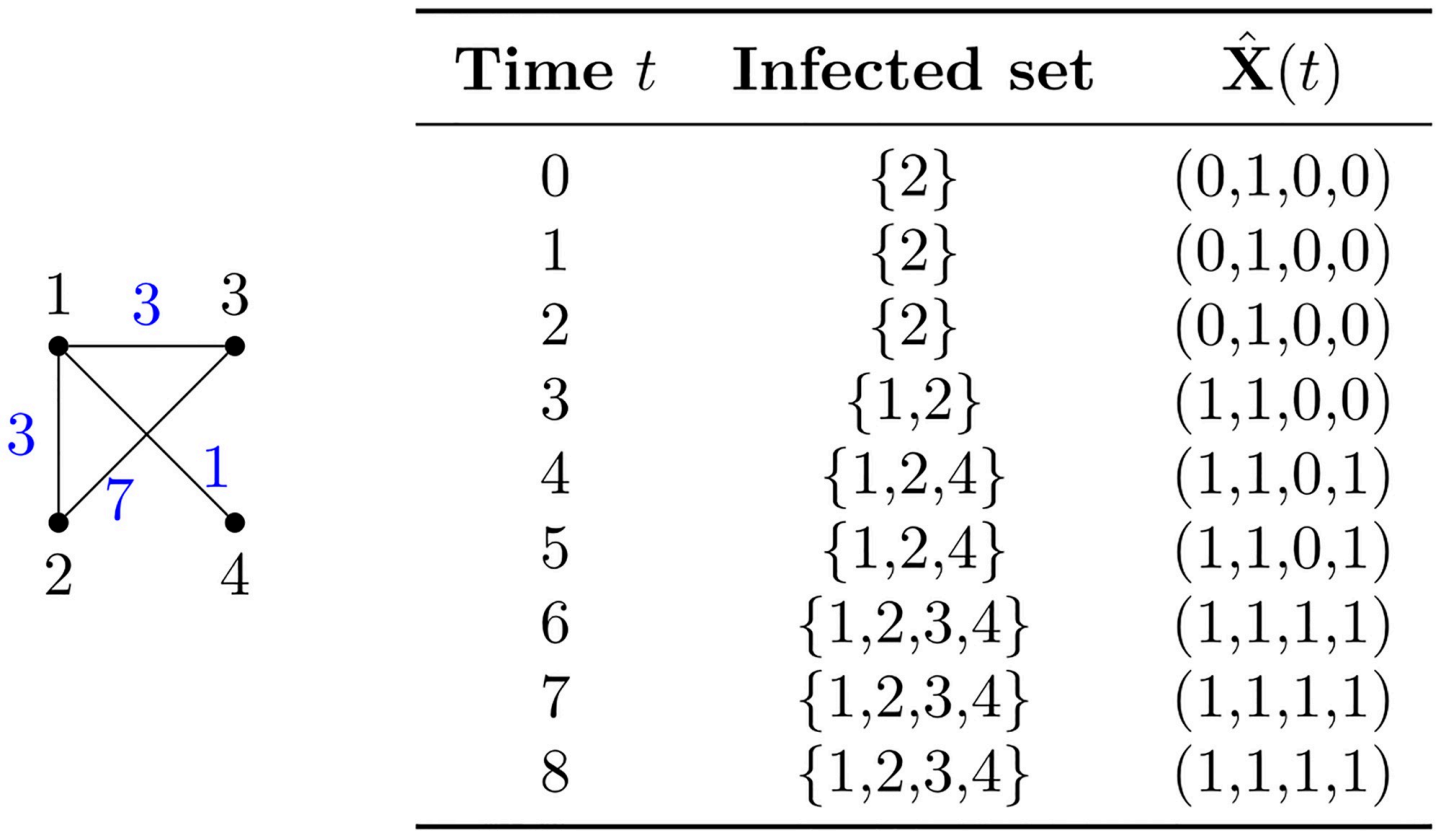
An example of the process $\hat{X}\left(t\right)$. A random choice of the potential infection times *τ*_*i*,*j*_ in our example graph, displayed in blue, and the resulting infection dynamic starting from $\hat{X}\left(0\right)=s_{2}$. Node 1 gets infected at time 3, node 4 gets infected at time 3 + 1 = 4, and node 3 gets infected at time 3 + 3 = 6 via node 1. The infection does not spread across edge {2, 3}, since both endpoints are already infected at time 7 when the potential infection is due to happen.

The Markov process that describes the infected individuals at time *t* in this construction is thus $\hat{X}\left(t\right)=s_{\left\{v\in V:\text{dist}(A,v)\leq t\right\}}$, where dist(*A*, *v*) = min_*w*∈*A*_dist(*w*, *v*). An example of possible sample values of the potential infection times *τ*_*i*,*j*_ and the resulting dynamic of the process $\hat{X}\left(t\right)$ is depicted in Fig 5.

The following proposition shows that this alternative construction provides the same result. This result could be considered intuitive by some, but we provide a formal proof.

**Theorem 4.4.** *The stochastic processes* **X**(*t*) *and* $\hat{X}\left(t\right)$ (*with the same initial state* $X\left(0\right)=\hat{X}\left(0\right)=s_{A}$) *are equidistributed*.

*Proof.* We shall show that the transition probabilities from $\hat{X}\left(t\right)$ to $\hat{X}\left(t+1\right)$ coincide with those in (6) and are independent of previous history. Let us condition on the state $\hat{X}\left(t\right)=x$, and consider the probability that $\hat{X}\left(t+1\right)=y$ has its *j*^th^ component equal to 0, i.e., that dist(*A*, *v*_*j*_) > *t* + 1. If *x*_*j*_ = 1, then dist(*A*, *v*_*j*_) ≤ *t*, hence it is impossible (probability 0) that this distance is larger than *t* + 1. Otherwise, let us call *n*_*j*_ the number of edges that join *v*_*j*_ with another node *v*_*i*_ with *x*_*i*_ = 1. For *v*_*j*_ to stay un-infected, it must be the case that dist(*A*, *v*_*i*_)+ *τ*_*i*,*j*_ > *t* + 1 for each such edge; and since we are assuming that *x*_*j*_ = 0 it must already be the case that dist(*A*, *v*_*i*_) + *τ*_*i*,*j*_ > *t*. By the memoryless property of the geometric distribution,

$$P[\tau_{i,j}>t-\text{dist}\left(A,v_{i}\right)+1|\tau_{i,j}>t-\text{dist}\left(A,v_{i}\right)]=1-\beta ,$$

and *τ*_*i*,*j*_ is independent from the potential infection times of all other edges by construction. Hence

$$P\left[{\hat{X}}_{j}\left(t+1\right)=0|{\hat{X}}_{i}\left(t\right)=0\right]={\left(1-\beta \right)}^{n_{j}}.$$

Hence we have proved the last two cases in (5); the first two cases follow by difference. The product formula (6) follows from the fact that each factor depends on different potential infection times *τ*_*i*,*j*_, which are independent.

The alternate construction leads immediately to an algorithm for more efficient Monte Carlo simulation of the system, which we describe in Algorithm 2. Some remarks are in order.

- The most expensive part of Algorithm 2 is the all-pairs shortest-path matrix computation, for a total cost of *O*(*N*|*V*||*E*|) using the algorithm in [40] where *N* is the number of simulations. We report in Section 4.3 on how the computation time compares with both the exact computation of (8) and the Monte Carlo simulations of Algorithm 1, and also discuss accuracy and precision.
- As the *M*^(*k*)^ are repeated samplings of a random variable, the central limit theorem (see, e.g., [25, Theorem 4.4.1]) applies, hence the estimate ${\hat{M}}_{inf}$ computed with this algorithm is affected by an error $|{\left({\hat{M}}_{inf}\right)}_{ij}-{\left(M_{inf}\right)}_{ij}|$ that scales as $\frac{\sigma_{ij}}{\sqrt{N}}$, where *σ*_*ij*_ is the standard deviation of the infection time from *i* to *j*. This standard deviation can in turn be estimated using the computed samplings, providing an estimate of the error.
- With the same strategy one can estimate not only the MICT ***μ***, but any quantity that depends only on the infection times; for instance, the mean time after which a given percentage *p*% of the nodes becomes infected.
- The construction that leads to Algorithm 2 can be generalized easily to directed graphs, and to deal with edge-dependent infection probabilities *β*_*i*,*j*_ (as long as they are independent from one another). In fact, the construction can also be generalized to any distribution of the potential infection times *τ*_*i*,*j*_, not necessarily a geometric one. This provides an avenue to incorporate a large number of extensions into this simple model, from superspreaders to SIR. For instance, a disease with a recovery time of *T* can be modeled by choosing a distribution such that $P[\tau_{i,j}>T]=0$.
- From this alternate description, one can prove easily the following result, which is a discrete-time analogue of part of [39, Theorem 2.3].

**Corollary 4.5** *On an undirected graph (possibly with edge-dependent infection probabilities* *β*_*i*,*j*_), *the MIT matrix* *M*_*inf*_ *is symmetric*.

*proof*. The MIT matrix *M*_*inf*_ is the mean over all possible samples $\tau_{i,j}^{\left(k\right)}$ of the sample MIT matrix *M*^(*k*)^, which is symmetric for each *k*.

We remark that in general for random walks on graphs, the mean first passage matrix is typically not symmetric (i.e. *m*_*i*,*j*_ ≠ *m*_*j*, *i*_), further delineating the contrast between mean first passage times obtained from simple random walks, and the mean infection times developed here.

**Algorithm 2**: Efficient Monte Carlo estimation of the mean infection time matrix *M*_*inf*_ (with *M*_*i*,*j*_ = *μ*({*v*_*i*_}, {*v*_*i*_, *v*_*j*_})) and MICT vector **m** with *m*_*i*_ = *μ*({*v*_*i*_}, *V*).

**Data**: A graph *G* = (*V*, *E*); infection probability *β*; a number of samples *N*.

**Result**: Estimates of the MIT matrix $M_{inf}\in R^{n\times n}$, and of the MICT vector $m=\left[m_{1},m_{2},\ldots ,m_{n}\right]\in R^{n}$.

*M*_*inf*_ ← 0; **m** ← **0**;

**for** *k* = 1, 2, …, *N* **do**; // Possibly in parallel

 **for** {*v*_*i*_, *v*_*j*_} ∈ *E* **do**

  Generate a random sample $\tau_{i,j}^{\left(k\right)}$ from a geometric distribution with parameter *β*;

 **end**

 Compute the all-pairs shortest-path matrix *M*^(*k*)^ of the graph (*V*, *E*) with weights $\tau_{i,j}^{\left(k\right)}$;

 Compute row-by-row maxima $m_{i}^{\left(k\right)}={\text{max}}_{j}M_{i,j}^{\left(k\right)}$;

 *M*_*inf*_ ← *M*_*inf*_ + *M*^(*k*)^; **m** ← **m**+ **m**^(*k*)^;

**end**

*M*_*inf*_ ← *M*_*inf*_/*N*; **m** ← **m**/*N*;

### 4.3 Accuracy and computational burden of the sampling strategy

For actual computation of mean infection times for a network, we have to deal with a transition matrix whose order is exponential with respect to the number of vertices of the network, as seen in (8). The computation is only feasible up to graphs of order *n* ≈ 15, due to storage problems. So, it is necessary to turn to algorithms to estimate MITs. Hence, we compare the two Monte Carlo estimations for mean infection times, in terms of speed and accuracy.

In order to see which algorithm is more accurate, we need to have the actual MITs, so we shall consider various graphs on at most 12 vertices. For each of the two algorithms, we report in Table 1 the mean *μ* and the variance *σ*^2^ of relative errors for entries between the actual and estimated MIT matrices, running 300 simulations.

**Table 1:** Accuracy for Algorithms 1 and 2.

Graphs	$\left(\mu_{1},\sigma_{1}^{2}\right)$	$\left(\mu_{2},\sigma_{2}^{2}\right)$
Fig 1	(0.0376, 0.0008)	(0.0333, 0.0005)
Paley graph of order 9	(0.0264, 0.0006)	(0.0214, 0.0003)
Petersen graph	(0.0241, 0.0005)	(0.0278, 0.0005)
Star of order 12	(0.0385, 0.0008)	(0.0229, 0.0003)
*L*(3, 4)	(0.0239, 0.0004)	(0.0155, 0.0002)

$\left(\mu_{1},\sigma_{1}^{2}\right)$ corresponds to Algorithm 1 and $\left(\mu_{2},\sigma_{2}^{2}\right)$ corresponds to Algorithm 2.

We now compare running times. The running time of Algorithm 1 mainly depends on the infection probability—that is, the smaller *β* is, the longer each simulation of the time taken for an individual *j* to become infected takes. In contrast, the most expensive part of Algorithm 2 is to compute the all-pairs shortest-path matrix, which does not depend on infection probability. For demonstration, we pick four contact networks [41], which are available at https://networkrepository.com. When a network is disconnected, we choose its largest component. In Table 2, we measure the computation times of the MIT matrix, running 100 simulations/samplings with different infection probabilities 0.2, 0.1, and 0.05.

**Table 2:** Comparison of running times for Algorithms 1 and 2.

Networks	(|*V*|, |*E*|)	$\left(t_{1}^{\left(0.2\right)},t_{2}^{\left(0.2\right)}\right)$	$\left(t_{1}^{\left(0.1\right)},t_{2}^{\left(0.1\right)}\right)$	$\left(t_{1}^{\left(0.05\right)},t_{2}^{\left(0.05\right)}\right)$
*G*_1_	(92, 755)	(8.56, 0.0677)	(15.30, 0.0838)	(29.93, 0.0844)
*G*_2_	(113, 2196)	(25.87, 0.1076)	(48.97, 0.1267)	(95.02, 0.1111)
*G*_3_	(274, 2124)	(433.8, 0.2370)	(911.2, 0.3281)	(1849.5, 0.3014)
*G*_4_	(327, 5818)	(191, 0.5188)	(344.8, 0.6023)	(647, 0.6193)

The graphs *G*_1_, *G*_2_, *G*_3_ and *G*_4_ correspond to ‘ia-workplace-contacts’, ‘ia-contacts_hypertext2009’, ‘ia-contact’ and ‘contacts-prox-high-school-2013’, respectively. We use $\left(t_{1}^{\left(p\right)},t_{2}^{\left(p\right)}\right)$ to indicate a pair of times in seconds for computing MIT matrix by 100 simulations/samplings with Algorithms 1 and 2 respectively, where *p* is the infection probability.

Table 2 shows that the approach based on the sampling strategy in Algorithm 2 is very efficient and scales very well with the size of the population of individuals, thus paving the way for a Markovian analysis of epidemic networks that does not suffer from the curse of dimensionality.

## 5 Exploring the ranking of nodes according to mean infection covering times

Ranking nodes in a graph has many applications in epidemic models, in terms of control strategies such as testing, vaccinating, and so on (see [6, 30, 33]). In this section, we investigate the mean infection covering times as defined in 4.2 and how they can be used as a measure of how pivotal each node is in the network to the spread of disease. The mean infection covering time of *v*_*i*_ is calculated using (8) and the observations following it as

$$\begin{array}{c} m_{i}=\mu_{\{v_{i}\},V}=e_{s_{v_{i}}}^{⊤}{\left(I-T_{\left({\bar{S}}_{v_{i}}\cup s_{V}\right)}\right)}^{-1}1. \end{array}$$ (10)

Then, *m*_*i*_ indicates the expected time for all individuals to get infected, starting from an initially infected node *v*_*i*_. We note that the author of [12] investigated the mean infection time for the case that the infection probability is 1. Moreover, as an analogous notion of the mean infection times, one may consider the *cover time* of a Markov chain [42], which is the expected value of the first time at which all the states have been visited.

### 5.1 Comparison with other indicators on 2-community networks

Here we compare what nodes are regarded as ‘most important’ according to different ranking indicators. In many social networks, node degree is an important metric to determine popular nodes, i.e., individuals who typically meet a large number of other individuals during a day, or who have many social ties on online social platforms. At the same time, it is also known that in networks with strong community structures, immunization interventions targeted at individuals bridging communities (e.g., families, school classes, working environment) are more effective than those simply targeting highly-connected individuals [33]. The reason for this is that regardless of the number of contacts one has, an individual bridging communities may pass the virus from one community to another community, and cause the infection to spread to a fully-susceptible community.

In what follows, we shall consider four random-walk based indicators or centrality measures, namely, the mean infection covering times introduced in this section, Kemeny’s constant, random walk betweenness (RWB), and random walk centrality (RWC). Here we provide formulae for RWB and RWC; see (3) for the description of Kemeny’s constant.

The random walk betweenness of node *i* in a network, denoted *b*_*i*_, measures the ‘expected net number of times a random walk passes through vertex *i* on its way from a source vertex *s* to a target vertex *t*, averaged over all *s* and *t*’, which is given [32] by

$$\begin{array}{c} b_{i}=\frac{2}{\left|V|(|V|-1\right)}\sum_{s<t}I_{i}^{\left(st\right)}, \end{array}$$

where $I_{i}^{\left(st\right)}$ is the current flow through vertex *i* starting from vertex *s* and ending at vertex *t*. This method for computing random walk betweenness depends on considering the graph as an electrical network in which each edge has a resistance of 1, and calculating the flow through each vertex (see [32] for details). This is then shown to be equivalent to the random walk interpretation given above.

The random walk centrality of node *i* in a network quantifies how easily or how quickly a random walker arrives at *i* from elsewhere in the network [31], which is given [43] by the reciprocal of the so-called *accessibility index* *α*_*i*_ of node *i*:

$$\begin{array}{c} \alpha_{i}=\sum_{k\neq i}\pi_{k}m_{k,i} \end{array}$$

where *π*_*k*_ is the stationary distribution for node *k* and *m*_*k*, *j*_ is the mean first passage time from *k* to *i*. The accessibility index can be interpreted as the expected time for the random walker to arrive at node *i*, starting from a randomly-chosen node *j*, where this starting node is chosen with respect to the stationary distribution vector (i.e. with respect to the degree of the node). Since the random walk centrality of a node *i* is $\frac{1}{\alpha_{i}}$, a large value for RWC indicates that the vertex in question is highly central, where ‘centrality’ is understood in terms of random walks terminating at a target vertex.

Regarding Kemeny’s constant, we score important nodes according to how their removal from the network affects the value of Kemeny’s constant: $c_{i}=K\left(G^{\prime }\right)-K\left(G\right)$ where *G*′ is the graph obtained from *G* by removing node *i*. That is, the higher the increment in Kemeny’s constant after deletion of a node is, the more critical the node is (recall that large values of Kemeny’s constant correspond to graphs which are not well-connected).

In the comparison that follows, we estimate MICTs by Algorithm 2 with 300 samplings. For RWB, RWC and Kemeny’s constant, larger values of the indicator correspond to more important nodes, while the reverse occurs for MICTs (i.e., lower values of MICT correspond to more important nodes).

**Fig 6:**
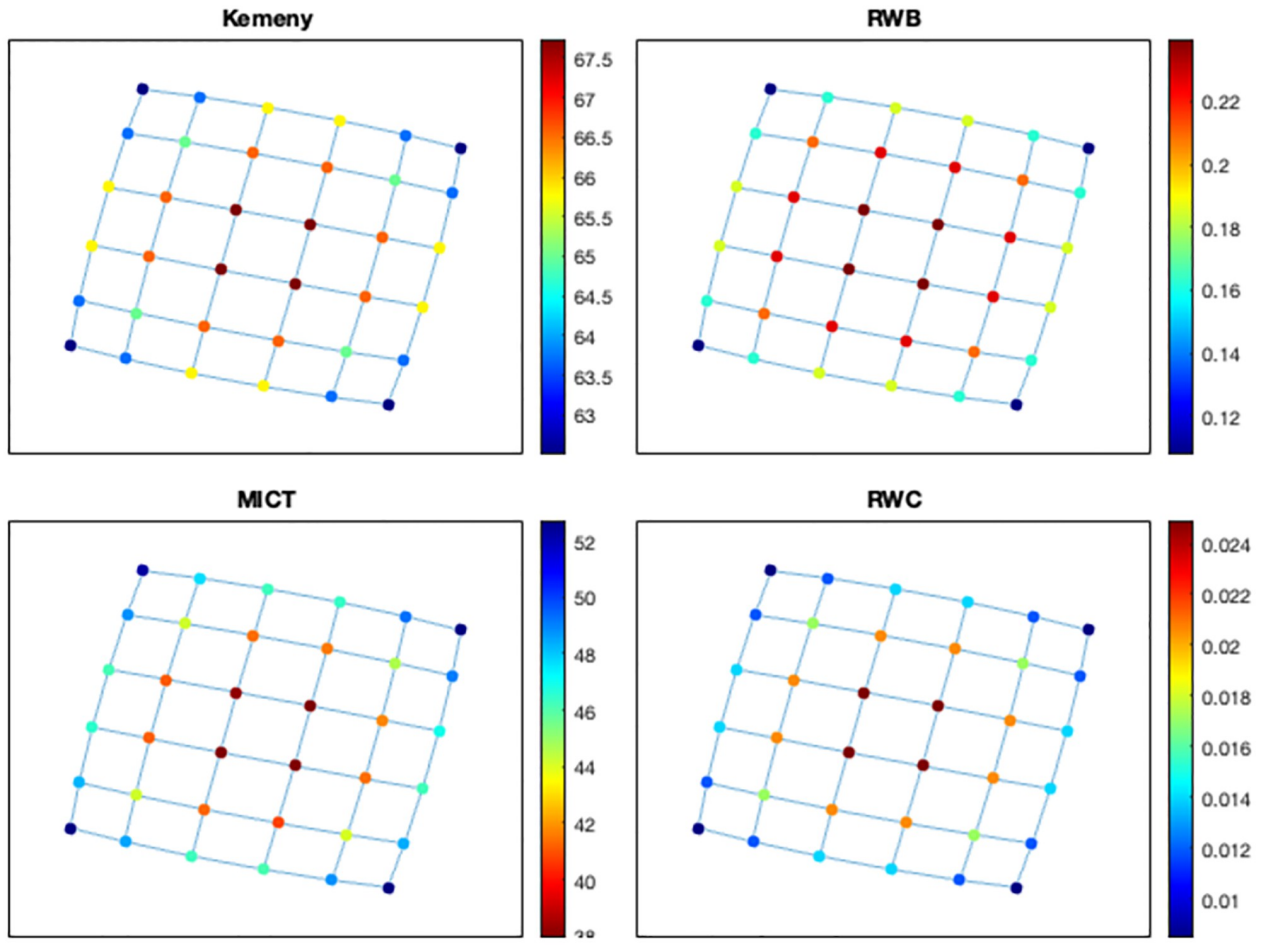
Ranking nodes in *L*(6, 6).

As a first example, consider the lattice graph *L*(6, 6). Fig 6 shows that all indicators agree that nodes placed in the center of the structure are the most important nodes.

**Fig 7:**
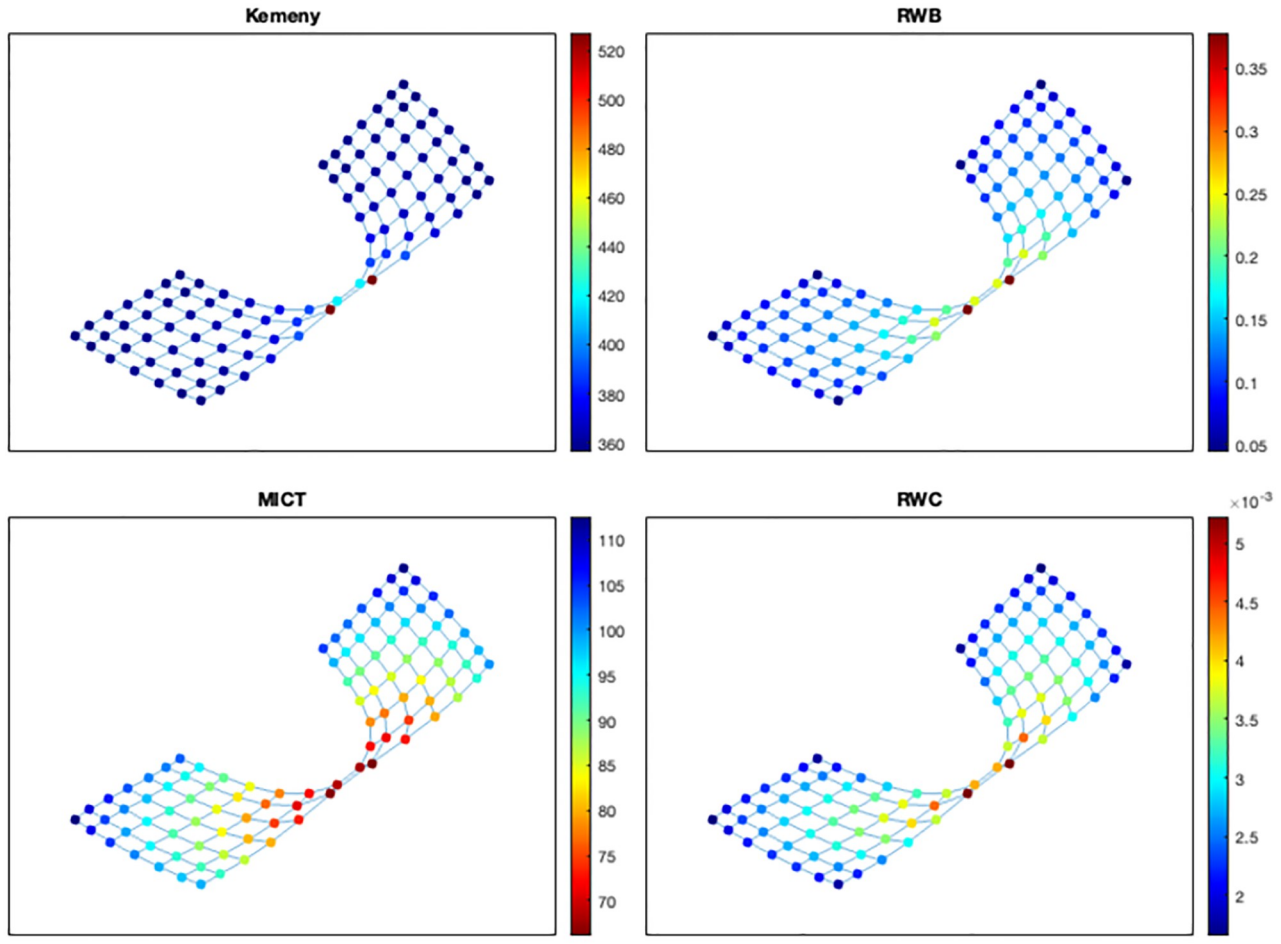
Ranking nodes in *G*(7, 7) based on the random walk-based indicators.

We now consider networks formed by two communities as follows: given two lattice graphs *L*(*n*_1_, *n*_1_) and *L*(*n*_2_, *n*_2_), we let an edge *e*_1_ join a vertex *v*_1_ at a corner of *L*(*n*_1_, *n*_1_) and a vertex *v*_2_ at a corner of *L*(*n*_2_, *n*_2_), and we also let *e*_2_ join a vertex adjacent to *v*_1_ in *L*(*n*_1_, *n*_1_) and a vertex adjacent to *v*_2_ in *L*(*n*_2_, *n*_2_). We denote by *G*(*n*_1_, *n*_2_) the resulting 2-community structure, and we shall refer to *e*_1_ and *e*_2_ as the community bridges. In particular, we now consider two types of 2-community structures: (1) two groups have the same size (i.e., *G*(7, 7)), and (2) one of two groups has a larger size than the other (i.e., *G*(10, 3)). Let us first consider the case of *G*(7, 7). As seen in Fig 7, all indicators show that the most critical nodes are positioned near the community bridges, and in particular, vertices *v*_1_ and *v*_2_ are the most critical ones. However, RWC indicates that the third and fourth ranked nodes are not incident to *e*_2_, while the other indicators do. Moreover, Kemeny’s constant suggests that nodes farther away from the community bridges have similar criticality scores of the nodes near the bridges; while MICTs provide more diversified scores that change gradually (in terms of criticality) as nodes are farther away from the bridges.

**Fig 8:**
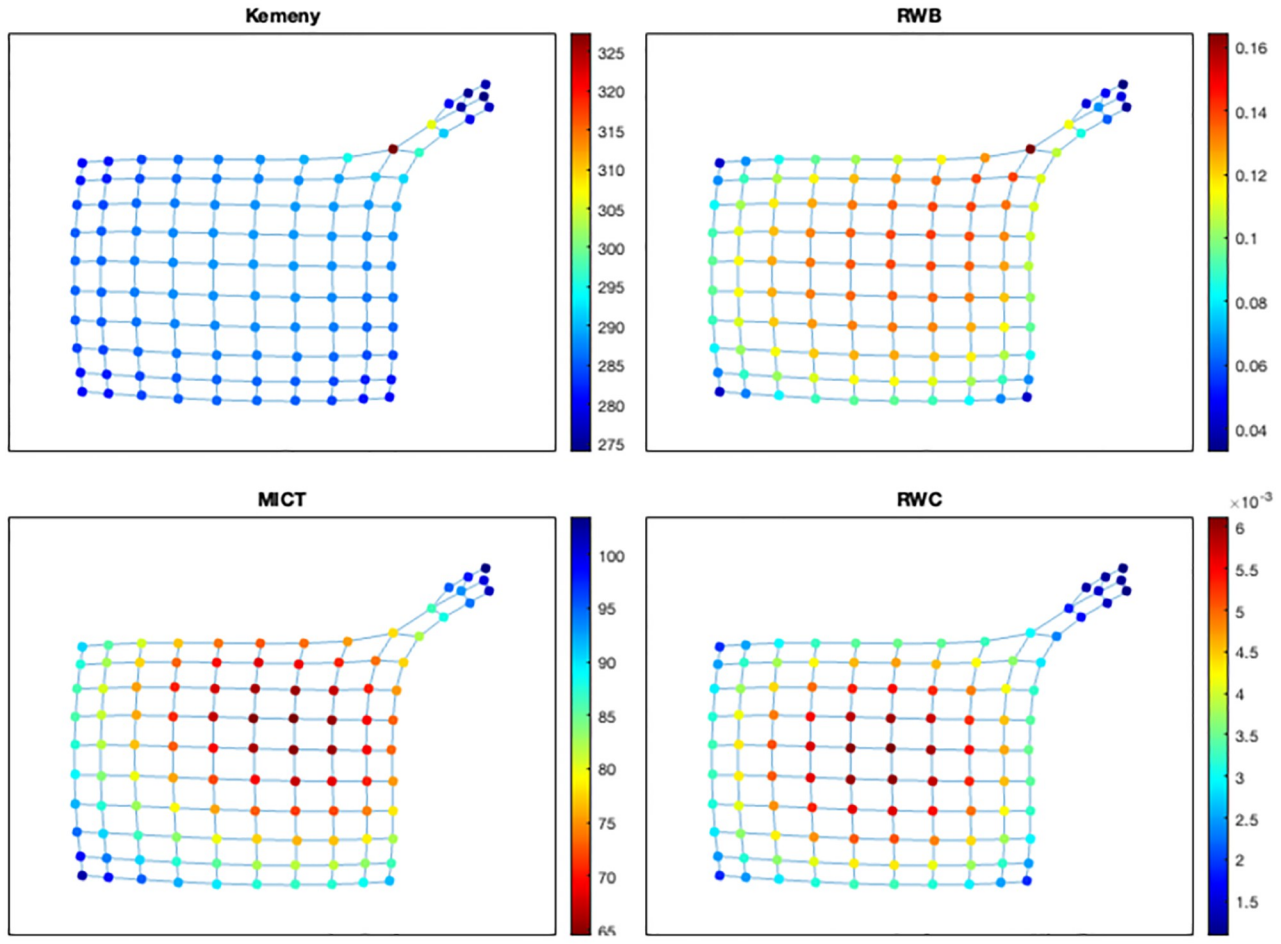
Ranking nodes in *G*(10, 3) based on the random walk-based indicators.

We finally consider the case of *G*(10, 3), shown in Fig 8. The four cases now provide different indications about what should be classified as the most critical nodes. In particular, the positions of the most critical nodes according to Kemeny’s constant, RWB, MICT, and RWC, respectively, vary in distance from the community bridges toward the central area of the larger community. This result appears to suggest that a criticality measure based on Kemeny’s constant via node removal could be useful for detecting community bridges regardless of the sizes of the groups in a community structure (which is in accordance with [6]). Conversely, RWC appears to rank highly the nodes that lie in the central area of the relatively larger community. MICT and RWB provide intermediate indications that take into account both the sizes of the groups and the presence of bridges (where RWB gives more importance to the community bridges than MICT). Finally, the values of the scores from MICT are spread more evenly across their (linear) scale.

### 5.2 Comparison with other indicators on a contact network

In this subsection, we present a contact network ‘ia-infect-dublin’ with 410 nodes and 2765 edges (available at https://networkrepository.com), and see how the random walk-based indicators suggest critical nodes based on our understanding in the previous section. Instead of colouring nodes according to indicators’ values, we display graphs with colouring by their own rankings. For MICT computations, we ran 300 simulations. Regarding computation times, it takes approximately 32.5, 1.5, 0.15, and 52.8 seconds for ranking nodes based on Kemeny’s constant, MICT, RWC, and RWB, respectively. This suggests that node centrality for Kemeny’s constant and RWB would be infeasible as the number of nodes in a network is larger.

**Fig 9:**
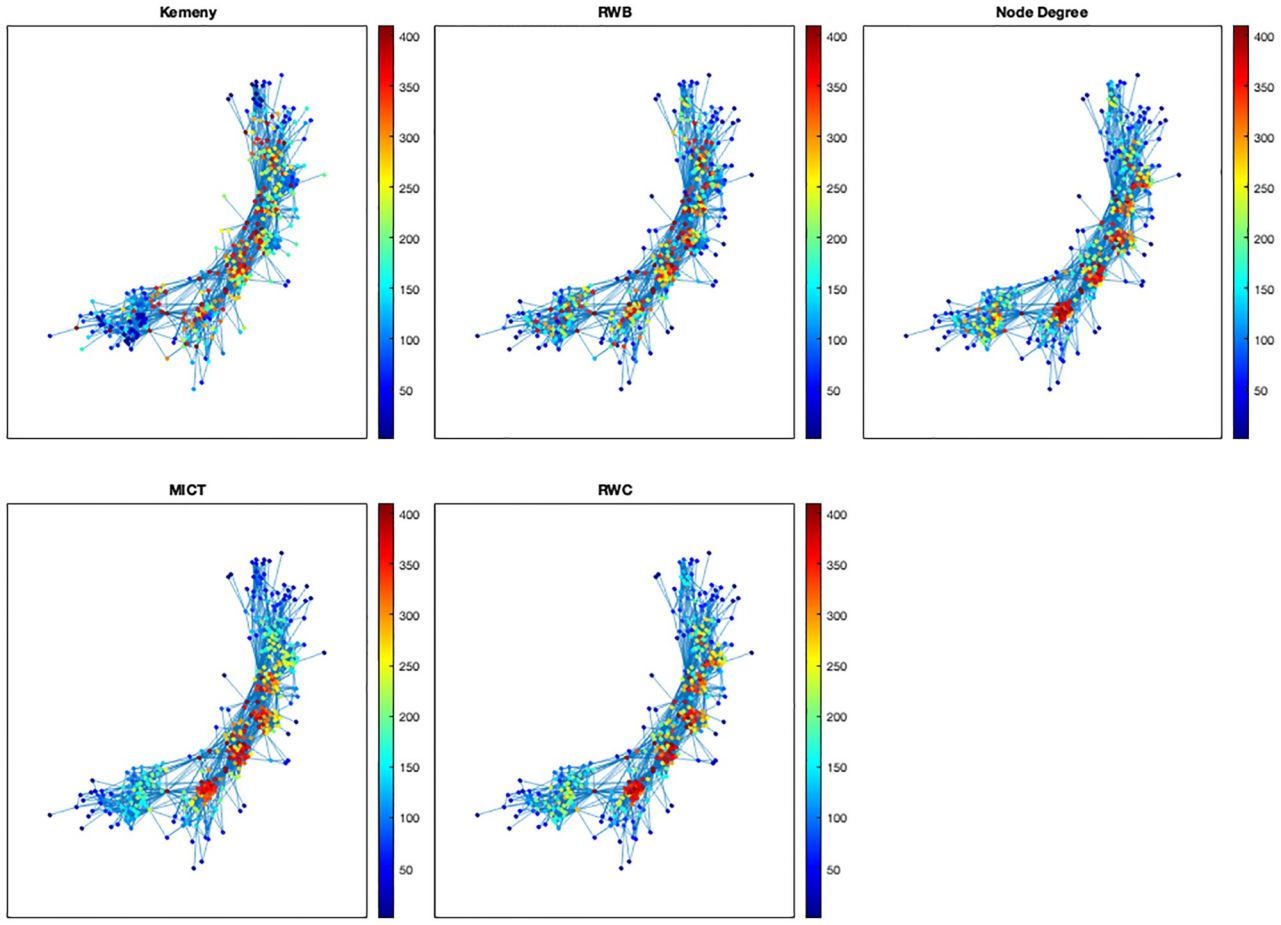
Comparison of ranking nodes based on the random walk-based indicators and node degrees in the contact network ‘ia-infect-dublin’.

As discussed in the previous section with 2-community structures, and indeed as seen in Fig 9, MICT and RWC are inclined to highlight important nodes in the central area rather than nodes nearby community bridges, while Kemeny’s constant and RWB are not. As a result, red-coloured nodes for MICT and RWC are more clustered in the central area while ones for Kemeny’s constant and RWB scatter around community bridges.

We can understand their different behaviours between Kemeny’s constant and RWB, and between MICT and RWC, considering communities in peripheral areas. Consider the community at the bottom left. Then, we can see that Kemeny’s constant does not suggest nodes in the centre of the community as important ones while RWB does, for the reason that the Kemeny-based indicator is the most affected by community bridges as seen in Fig 8.

Consider two communities at the bottom left and the top right. We can see that MICT ranks less important nodes in the communities than RWC does. Since RWC is the least affected by community bridges among the random walk-based indicators from Fig 9, one can interpret the difference in that MICT ranks more important nodes in the central area because they have more community bridges.

Finally, since one might expect that the result of rankings from MICT is relevant to node degrees, we consider ranking nodes according to node degrees. Its result is the most similar to the one from RWC.

## 6 Conclusions and future work

Random walks on graphs have been widely used in the literature to analyse and predict epidemic spreads, and to plan, design and compare the outcomes of different mitigation policies in terms of their ability to control the spreading of a virus. As we show in this manuscript, simplistic models based on single random walkers are very convenient in terms of computational aspects, and thus have a significant predictive power in networks of realistic size. However, they are not consistent with the actual dynamics of the virus, in terms of the number of steps which is actually required before the virus spreads from one individual to another individual in the network. Conversely, other models accurately depict the actual spreading of the virus, also in terms of expected steps for transmission, but grow exponentially with the number of individuals, and thus are not feasible for accurate predictions in large networks, unless special symmetries in the graph reduce the computational burden.

In this work we thoroughly review both the aforementioned models, and we describe a sampling strategy that allows one to adopt the more accurate model, in terms of expected steps for infection, to also analyse larger graphs without incurring in an exponential growth of the state space. In addition, we introduce a novel indicator of criticality of nodes, which is based on the expected number of steps for infection to reach the entire population. A comparison of the properties of this indicator with other similar importance indicators is also provided.

Our work can be extended in a number of directions. First of all, while all the results we have provided have been obtained from a simple SI model, where individuals are either infected or susceptible, the same approach can be easily generalized to more sophisticated, and realistic, epidemic models where also recovered, exposed, or individuals at other stages of the infection, are considered. In these cases, we are interested in ascertaining whether the proposed random-walk based model, which is now evaluable also for larger networks, does provide further insights in the dynamics of a virus. In particular, it would be interesting to study if there is a correlation between the identified critical nodes via MICTs, and actual superspreaders and superspreading events in real networks, which are known to having played an important role in the spreading of a virus [44–46]. Finally, computational aspects, convergence to the actual MICTs, and analytical tight bounds on the covering times are all aspects which still partially remain open questions, and we are interested in addressing in the future.

## References

[1] Langville AN, Meyer CD. Google’s PageRank and Beyond—The Science of Search Engine Rankings. Princeton, NJ: Princeton University Press; 2006.

[2] Yan J, He H, Sun Y. Integrated Security Analysis on Cascading Failure in Complex Networks. IEEE Transactions on Information Forensics and Security. 2014;9(3):451–463. doi: 10.1109/TIFS.2014.2299404

[3] Sporns O. Graph theory methods: applications in brain networks. Dialogues in Clinical Neuroscience. 2018;20(2):111–121. doi: 10.31887/DCNS.2018.20.2/osporns 30250388 PMC6136126

[4] Vespignani A. Modelling dynamical processes in complex socio-technical systems. Nature Physics. 2012;8:32–39. doi: 10.1038/nphys2160

[5] Crisostomi E, Kirkland S, Shorten R. A Google-like model of road network dynamics and its application to regulation and control. International Journal of Control. 2011;84(3):633–651. doi: 10.1080/00207179.2011.568005

[6] Yilmaz S, Dudkina E, Bin M, Crisostomi E, Ferraro P, Murray-Smith R, et al. Kemeny-based testing for COVID-19. PLoS One. 2020;15(11):e0242401. doi: 10.1371/journal.pone.0242401 33211725 PMC7676669

[7] Levene M, Loizou G. Kemeny’s constant and the random surfer. The American mathematical monthly. 2002;109(8):741–745. doi: 10.2307/3072398

[8] Estrada E. The structure of complex networks: theory and applications. Oxford University Press; 2012.

[9] Liu Z, Emad N, Amor SB, Lamure M. A parallel IRAM algorithm to compute PageRank for modeling epidemic spread. In: 2013 25th International Symposium on Computer Architecture and High Performance Computing. IEEE; 2013. p. 120–127.

[10] Shams B, Khansari M. On the impact of epidemic severity on network immunization algorithms. Theoretical Population Biology. 2015;106:83–93. doi: 10.1016/j.tpb.2015.10.007 26505554 PMC7126281

[11] Chu A, Huber G, McGeever A, Veytsman B, Yllanes D. A random-walk-based epidemiological model. Scientific reports. 2021;11(1):1–11. doi: 10.1038/s41598-021-98211-5 34588487 PMC8481482

[12] Dimitriou T, Nikoletseas S, Spirakis P. The infection time of graphs. Discrete Applied Mathematics. 2006;154(18):2577–2589. doi: 10.1016/j.dam.2006.04.026

[13] Giakkoupis G, Mallmann-Trenn F, Saribekyan H. How to spread a rumor: Call your neighbors or take a walk? In: Proceedings of the 2019 ACM Symposium on Principles of Distributed Computing; 2019. p. 24–33.

[14] Ajelli M, Gonçalves B, Balcan D, Colizza V, Hu H, Ramasco JJ, et al. Comparing large-scale computational approaches to epidemic modeling: agent-based versus structured metapopulation models. BMC infectious diseases. 2010;10(1):1–13. doi: 10.1186/1471-2334-10-190 20587041 PMC2914769

[15] Aleta A, Martin-Corral D, Pastore y Piontti A, Ajelli M, Litvinova M, Chinazzi M, et al. Modelling the impact of testing, contact tracing and household quarantine on second waves of COVID-19. Nature Human Behaviour. 2020;4(9):964–971. doi: 10.1038/s41562-020-0931-9 32759985 PMC7641501

[16] Chang S, Pierson E, Koh PW, Gerardin J, Redbird B, Grusky D, et al. Mobility network models of COVID-19 explain inequities and inform reopening. Nature. 2021;589(7840):82–87. doi: 10.1038/s41586-020-2923-3 33171481

[17] Eubank S, Guclu H, Anil Kumar V, Marathe MV, Srinivasan A, Toroczkai Z, et al. Modelling disease outbreaks in realistic urban social networks. Nature. 2004;429(6988):180–184. doi: 10.1038/nature02541 15141212

[18] Grassberger P. Two-dimensional SIR epidemics with long range infection. Journal of statistical physics. 2013;153(2):289–311. doi: 10.1007/s10955-013-0824-7

[19] Lam H, Liu Z, Mitzenmacher M, Sun X, Wang Y. Information dissemination via random walks in d-dimensional space. In: Proceedings of the Twenty-Third Annual ACM-SIAM Symposium on Discrete Algorithms. SIAM; 2012. p. 1612–1622.

[20] Pettarin A, Pietracaprina A, Pucci G, Upfal E. Infectious random walks. arXiv preprint arXiv:10071604. 2010;.

[21] Kemeny J, Snell J. Finite Markov Chains. Springer-Verlag, New York-Heidelberg; 1976.

[22] Kirkland S, Zeng Z. Kemeny’s constant and an analogue of Braess’ paradox for trees. The Electronic Journal of Linear Algebra. 2016;31:444–464. doi: 10.13001/1081-3810.3222

[23] Hunter JJ. Mixing times with applications to perturbed Markov chains. Linear Algebra and its Applications. 2006;417(1):108–123. doi: 10.1016/j.laa.2006.02.008

[24] Kirkland S. Fastest expected time to mixing for a Markov chain on a directed graph. Linear Algebra and its Applications. 2010;433(11-12):1988–1996. doi: 10.1016/j.laa.2010.07.016

[25] Norris JR. Markov Chains. Cambridge Series in Statistical and Probabilistic Mathematics. Cambridge University Press; 1997.

[26] Daley DJ, Gani J. Epidemic modelling: an introduction. 15. Cambridge University Press; 2001.

[27] G’enois M, Barrat A. Can co-location be used as a proxy for face-to-face contacts? EPJ Data Science. 2018;7(1):11. doi: 10.1140/epjds/s13688-018-0140-1

[28] D’Ambrosio R, Giordano G, Mottola S, Paternoster B. Stiffness analysis to predict the spread out of fake information. Future Internet. 2021;13(9):222. doi: 10.3390/fi13090222

[29] Shrivastava G, Kumar P, Ojha RP, Srivastava PK, Mohan S, Srivastava G. Defensive modeling of fake news through online social networks. IEEE Transactions on Computational Social Systems. 2020;7(5):1159–1167. doi: 10.1109/TCSS.2020.3014135

[30] Dudkina E, Bin M, Breen J, Crisostomi E, Ferraro P, Kirkland S, et al. On node ranking in graphs. arXiv preprint arXiv:210709487. 2021;.

[31] Noh JD, Rieger H. Random walks on complex networks. Physical review letters. 2004;92(11):118701. doi: 10.1103/PhysRevLett.92.118701 15089179

[32] Newman ME. A measure of betweenness centrality based on random walks. Social networks. 2005;27(1):39–54. doi: 10.1016/j.socnet.2004.11.009

[33] Salathé M, Jones JH. Dynamics and control of diseases in networks with community structure. PLoS computational biology. 2010;6(4):e1000736. doi: 10.1371/journal.pcbi.1000736 20386735 PMC2851561

[34] Ahn HJ, Hassibi B. On the mixing time of the SIS Markov chain model for epidemic spread. In: 53rd IEEE Conference on Decision and Control. IEEE; 2014. p. 6221–6227.

[35] Ganesh A, Massoulié L, Towsley D. The effect of network topology on the spread of epidemics. In: Proceedings IEEE 24th Annual Joint Conference of the IEEE Computer and Communications Societies. vol. 2. IEEE; 2005. p. 1455–1466.

[36] Van Mieghem P, Omic J, Kooij R. Virus spread in networks. IEEE/ACM Transactions On Networking. 2008;17(1):1–14. doi: 10.1109/TNET.2008.925623

[37] Ding N, Williams S, Liu Y, Li XS. Leveraging One-Sided Communication for Sparse Triangular Solvers. In: Proceedings of the 2020 SIAM Conference on Parallel Processing for Scientific Computing. SIAM; 2020. p. 93–105.

[38] Buluç A, Fineman JT, Frigo M, Gilbert JR, Leiserson CE. Parallel sparse matrix-vector and matrix-transpose-vector multiplication using compressed sparse blocks. In: Proceedings of the twenty-first annual symposium on Parallelism in algorithms and architectures; 2009. p. 233–244.

[39] Goering M, Albin N, Poggi-Corradini P, Scoglio CM, Sahneh FD. Numerical investigation of metrics for epidemic processes on graphs. In: Matthews MB, editor. 49th Asilomar Conference on Signals, Systems and Computers, ACSSC 2015, Pacific Grove, CA, USA, November 8-11, 2015. IEEE; 2015. p. 1317–1322. Available from: 10.1109/ACSSC.2015.7421356.

[40] Thorup M. Undirected single-source shortest paths with positive integer weights in linear time. J ACM. 1999;46:362–394. doi: 10.1145/316542.316548

[41] Rossi RA, Ahmed NK. The Network Data Repository with Interactive Graph Analytics and Visualization. In: AAAI; 2015. Available from: http://networkrepository.com.

[42] Levin DA, Peres Y. Markov chains and mixing times. vol. 107. American Mathematical Soc.; 2017.

[43] Kirkland S. Random walk centrality and a partition of Kemeny’s constant. Czechoslovak Mathematical Journal. 2016;66(3):757–775. doi: 10.1007/s10587-016-0291-9

[44] Aleta A, Martín-Corral D, Bakker MA, Pastore y Piontti A, Ajelli M, Litvinova M, et al. Quantifying the importance and location of SARS-CoV-2 transmission events in large metropolitan areas. Proceedings of the National Academy of Sciences. 2022;119(26):e2112182119. doi: 10.1073/pnas.2112182119 35696558 PMC9245708

[45] Althouse BM, Wenger EA, Miller JC, Scarpino SV, Allard A, Hébert-Dufresne L, et al. Superspreading events in the transmission dynamics of SARS-CoV-2: Opportunities for interventions and control. PLoS biology. 2020;18(11):e3000897. doi: 10.1371/journal.pbio.3000897 33180773 PMC7685463

[46] Kawagoe K, Rychnovsky M, Chang S, Huber G, Li L, Miller J, et al. Epidemic dynamics in inhomogeneous populations and the role of superspreaders. Physical Review Research. 2021;3(3):033283. doi: 10.1103/PhysRevResearch.3.033283

